# Significantly different clinical phenotypes associated with mutations in synthesis and transamidase+remodeling glycosylphosphatidylinositol (GPI)-anchor biosynthesis genes

**DOI:** 10.1186/s13023-020-1313-0

**Published:** 2020-02-04

**Authors:** Leigh C. Carmody, Hannah Blau, Daniel Danis, Xingman A. Zhang, Jean-Philippe Gourdine, Nicole Vasilevsky, Peter Krawitz, Miles D. Thompson, Peter N. Robinson

**Affiliations:** 1 The Jackson Laboratory for Genomic Medicine, 10 Discovery Drive, Farmington, CT 06032 USA; 2 Oregon Health & Science University, Portland, OR 97239 USA; 3 Institute of Genomic Statistics and Bioinformatics, University of Bonn, Bonn, Germany; 4 Department of Pediatrics, UCSD School of Medicine, La Jolla, CA 92093 USA; 5 Institute for Systems Genomics, University of Connecticut, Farmington, CT USA

**Keywords:** GPI-anchor, Glycosylphosphatidylinositols, Congenital disorders of glycosylation, Human phenotype ontology

## Abstract

**Background:**

Defects in the glycosylphosphatidylinositol (GPI) biosynthesis pathway can result in a group of congenital disorders of glycosylation known as the inherited GPI deficiencies (IGDs). To date, defects in 22 of the 29 genes in the GPI biosynthesis pathway have been identified in IGDs. The early phase of the biosynthetic pathway assembles the GPI anchor (Synthesis stage) and the late phase transfers the GPI anchor to a nascent peptide in the endoplasmic reticulum (ER) (Transamidase stage), stabilizes the anchor in the ER membrane using fatty acid remodeling and then traffics the GPI-anchored protein to the cell surface (Remodeling stage).

**Results:**

We addressed the hypothesis that disease-associated variants in either the Synthesis stage or Transamidase+Remodeling-stage GPI pathway genes have distinct phenotypic spectra. We reviewed clinical data from 58 publications describing 152 individual patients and encoded the phenotypic information using the Human Phenotype Ontology (HPO). We showed statistically significant differences between the Synthesis and Transamidase+Remodeling Groups in the frequencies of phenotypes in the musculoskeletal system, cleft palate, nose phenotypes, and cognitive disability. Finally, we hypothesized that phenotypic defects in the IGDs are likely to be at least partially related to defective GPI anchoring of their target proteins. Twenty-two of one hundred forty-two proteins that receive a GPI anchor are associated with one or more Mendelian diseases and 12 show some phenotypic overlap with the IGDs, represented by 34 HPO terms. Interestingly, GPC3 and GPC6, members of the glypican family of heparan sulfate proteoglycans bound to the plasma membrane through a covalent GPI linkage, are associated with 25 of these phenotypic abnormalities.

**Conclusions:**

IGDs associated with Synthesis and Transamidase+Remodeling stages of the GPI biosynthesis pathway have significantly different phenotypic spectra. GPC2 and GPC6 genes may represent a GPI target of general disruption to the GPI biosynthesis pathway that contributes to the phenotypes of some IGDs.

## Introduction

Glycosylphosphatidylinositols (GPIs) are glycolipids that act as membrane anchors of many cell surface proteins. The GPI-anchor biosynthesis pathway covalently attaches the glycolipid to the C-termini of nascent proteins as a post-translational modification [1]. Defects in this pathway represent a relatively new subclass of congenital disorders of glycosylation (CDG) termed *inherited GPI deficiencies* (IGDs), which are the result of mutations in one of nearly 30 genes that encode portions of the GPI biosynthetic pathway [2].

**Fig. 1:**
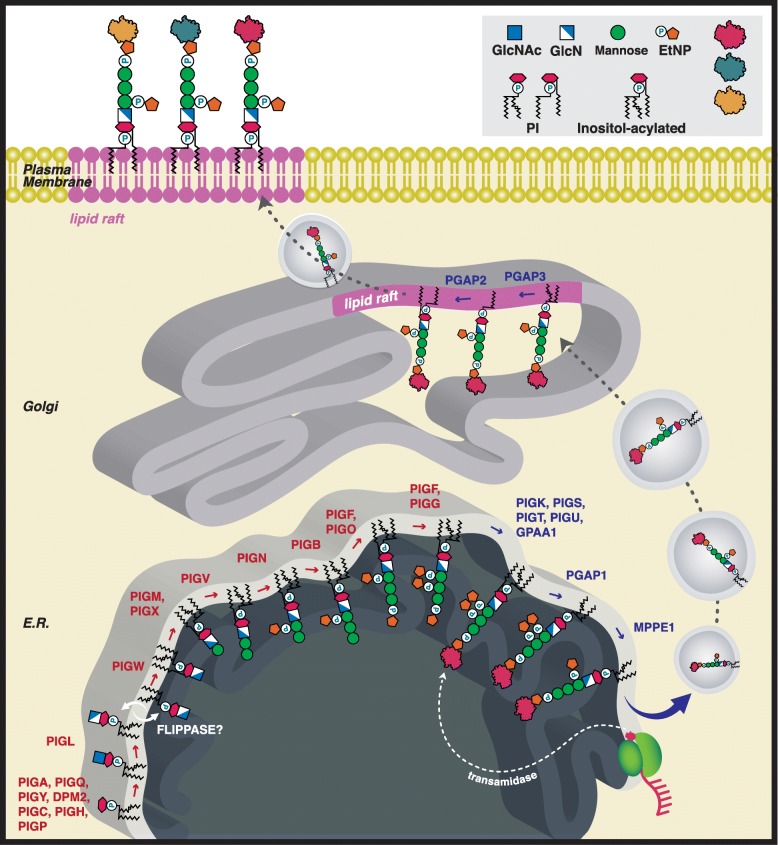
GPI biosynthesis pathway. Illustrated is the biosynthetic pathway of GPI-AP. In the Synthesis-stage, twenty genes are responsible for synthesizing the GPI anchor (Synthesis Group, genes highlighted in red). The Transamidase+Remodelinglate-stage couples the protein to the GPI anchor and mediates trafficking through the Golgi apparatus to the cell surface (Tranasmidase+Remodeling Group, genes highlighted in blue) [3–6]. Abbreviations: PI: phosphatidylinositol; EtNP: ethanolamine phosphate; GlcN: D-Glucosamine; GlcNAc: N-Acetyl-D-glucosamine; E.R.:endoplasmic reticulum. Gene symbols: See Tables 1 and 2

The GPI-anchor biosynthesis can be broken down into a Synthesis and a Transamidase+Remodeling stage (Fig. 1). The first or Synthesis stage results in the stepwise construction of the GPI anchor. The second or Transamidase+Remodeling-stage involves the transfer of a nascent peptide to the GPI anchor by the transamidase complex and results in fatty acid remodeling necessary to stabilize the anchor in the membrane. This happens possibly in conjunction with lipid raft formation before​ ​it​ ​is​ ​trafficked by secretory vesicles to the cell surface [7, 8]. Approximately 150 proteins are GPI anchored, including enzymes, structural molecules, receptors, and regulatory proteins [1]. Misregulation of the GPI-anchored proteins (GPI-AP), which occurs as a result of mutations in the GPI-biosynthesis pathway, leads to the variety of phenotypes observed in IGD.

IGD disorders are more common than initially recognized. A study of 4293 parent-child developmental disability trios suggested that IGDs alone account for 0.15% of all developmental disorders [9]. The IGD-associated disorders listed in Table 1 result from complete or partial inactivation of these GPI biosynthesis enzymes. The phenotypes that characterize these disorders often include seizures, intellectual disability, coarse facial features, hypotonia, microcephaly, hearing impairment, and joint contractures. The diseases also display phenotypic abnormalities of the skin, heart, urinary system, and skeleton, which are less common features [3]. Reduced surface levels of GPI-APs or abnormal GPI-AP structure are common in IGD [23].

**Table 1:** Genes in the GPI biosynthetic pathway anchoring process, Synthesis stage

Gene name (Entrez ID)	# Patients curated	Disease Association	Inheritance
DPM1 (8813)	3	OMIM:608799 CDG, Type Ie [10]	AR
DPM2 (8818)	3	OMIM:615042; CDG, Type Iu [11]	AR
DPM3 (54344)	2	OMIM:612937 CDG, Type Io, Muscular dystrophy-dystroglycanopathy (limb-girdle), type C, 15 [12]	AR
MPDU1 (9526)	1	OMIM:609180 CDG, Type If [13]	AR
PIGA (5277)	21	OMIM:300868 Multiple Congenital Anomalies-hypotonia-seizures Syndrome 2 (MCAHS 2); MIM:300818 Paroxysmal Nocturnal Hemoglobinuria 1 [14]	XLR
PIGB (9488)	0	n/a	
PIGC (5279)	3	OMIM:617816 GPI Biosynthesis Defect 16 [15]	AR
PIGF (5281)	0	n/a	
PIGG (54872)	5	OMIM:616917 Mental Retardation, Autosomal Recessive 53 [16]	AR
PIGH (5283)	1	OMIM: 618010 GPI Biosynthesis Defect 17 [17]	AR
PIGL (9487)	8	OMIM:280000 Chime Syndrome [18–20]	AR
PIGM (93183)	3	OMIM:610293 Glycosylphosphatidylinositol Deficiency [21]	AR
PIGN (23556)	11	OMIM:614080 MCAHS 1	AR
PIGO (84720)	9	OMIM:614749 HPMRS 2	AR
PIGP (51227)	2	OMIM:617599 Epileptic Encephalopathy, Early Infantile, 55 (EIEE 55) [22]	AR
PIGQ (9091)	1	Possible Association With Early Infantile Epileptic Encephalopathy (EIEE) [17, 18]	
PIGV (55650)	13	OMIM:239300 HPMRS 1	AR
PIGW (284098)	3	OMIM:616025 GPI Biosynthesis Defect 11	AR
PIGX (54965)	0	n/a	
PIGY (84992)	4	OMIM:616025 HPMRS 6	AR

*AR* Autosomal recessive, *AD* Autosomal dominant, *XLR* X-linked recessive. The Online Mendelian Inheritance in Man (OMIM) identifier for the disease is shown if available. Abbreviations: *GPI* Glycosylphosphatidylinositol, *HPMRS* Hyperphosphatasia With Mental Retardation Syndrome, *MCAHS* Multiple congenital anomalies-hypotonia-seizures, *CDG* Congenital Disorder Of Glycosylation, *EIEE* Early Infantile Epileptic Encephalopathy, *PNH* Paroxysmal Nocturnal Hemoglobinuria, *ARMR* Autosomal recessive mental retardation. “n/a” is entered in the Disease Association column if no such association has been identified to date

The first disease identified of the IGDs, hyperphosphatasia with mental retardation syndrome (HPMRS), is associated with variants in both Synthesis-stage and Transamidase+Remodeling-stage genes [24–29]. Specifically, HPMRS is caused by mutations in one of the four genes necessary for the biosynthesis of the GPI anchor in the endoplasmic reticulum (*PIGV*, *PIGO*, *PIGW,* and *PIGY*), or two genes necessary for the post-GPI attachment to proteins (*PGAP*) type 2 (*PGAP2*) and type 3 (*PGAP3*) [24, 30]. Multiple congenital anomalies-hypotonia-seizures (MCAHS) syndrome is a related disorder, although patients do not have hyperphosphatasia (persistently *elevated alkaline phosphatase*). MCAHS1 [31–35] results from inherited *PIGN* mutations, a critical gene within the GPI biosynthetic pathway [31, 32, 34]. There is considerable phenotypic variability in MCAHS1, probably reflecting the fact that there is residual GPI associated function [22, 33, 36]. Germline *PIGA* mutations give rise to an X-linked MCAHS2 [14, 37, 38], and somatic mutations in bone marrow cells result in paroxysmal nocturnal hemoglobinuria [39–41]. Finally, MCAHS3 syndrome results from the autosomal recessive inheritance of mutations in *PIGT* [42–45]. Especially at the severe end of the phenotypic spectrum of HPMRS, there is substantial phenotypic overlap with MCAHS [46]. Additional IGDs are not classified as either HPMRS or MCAHS have been identified. An overview of all IGD described at the time of this writing is provided in Tables 1 and 2.

**Table 2:** Genes in the GPI biosynthetic pathway anchoring process, Transamidase+Remodeling stage. Abbreviations as in Table 1

Gene name (Entrez ID)	# Patients curated	Disease Association	Inheritance
GPAA1 (8733)	10	OMIM:617810 GPI biosynthesis defect 15	AR
MPPE1 (65258)	0	n/a	
PGAP1 (80055)	7	OMIM: 615802 Mental retardation, autosomal recessive 42	AR
PGAP2 (27315)	10	OMIM: 614207 HPMRS 3	AR
PGAP3 (93210)	22	OMIM:615716 HPMRS 4	AR
PIGK (10026)	0	n/a	
PIGS (94005)	0	OMIM:618143 GPI biosynthesis defect-18	AR
PIGT (51604)	10	OMIM:615399, Paroxysmal nocturnal hemoglobinuria 2, (PNH 2); MIM: 615398 MCAHS 3	AD/AR
PIGU (128869)	0	n/a	

A recent review of the phenotypes from 202 IGD patients taken either from the literature or from in-house clinical data chronicled the wide array of phenotypes observed with mutations in each of the GPI-anchor biosynthesis pathway genes, including cognitive impairment, seizures, and congenital malformations [3]. Here, we identified published clinical case studies describing individuals with disease-causing variants in any gene coding for an enzyme in the GPI-anchoring biosynthesis pathway. We then compared phenotypic differences in the Synthesis and Transamidase+Remodeling stages of the GPI-anchoring pathway utilizing the Human Phenotype Ontology (HPO), a standardized vocabulary of phenotypic abnormalities [47]. We demonstrated a number of statistically significant differences in the phenotypic spectrum of diseases in the two groups, suggesting that differential effects on the biochemical function of the GPI pathway may result in different clinical manifestations. We reviewed the phenotypes of diseases caused by defects in individual GPI-anchored proteins and identified a number of candidate GPI-anchored proteins that could cause individual component phenotypes that characterize the IGDs.

## Results

In this work, we present a computational analysis to address the question of whether there are differences in the phenotypic spectrum of diseases associated with genes in the Synthesis and Transamidase+Remodeling phases of GPI anchor biosynthesis (Fig. 1). We first performed a comprehensive literature review of all published case reports about individuals diagnosed with diseases caused by variants in a GPI-anchor pathway gene. We then extracted the patient information, mutation(s) information, and all phenotypic data about each patient using terms from the HPO [48–50] (Tables 1 and 2). Clinical data from 58 publications were included in this study, comprising a total of 152 individual patients for whom detailed phenotypic descriptions were available, representing IGDs associated with a total of 22 genes involved in the GPI-biosynthesis pathway (Additional file 1: Table S1).

### Synthesis vs. Transamidase+Remodeling phenotypes

We divided the GPI biosynthesis pathway into Synthesis and Transamidase+Remodeling stages. Enzymes in the Synthesis Group mediate assembly of the GPI precursor backbone in the endoplasmic reticulum (ER) membrane. Enzymes in the Transamidase+Remodeling Group facilitate the coupling of the GPI to the C-terminus of a newly synthesized protein within the lumen of the ER, cleavage of a C-terminal GPI-addition signal peptide, and enable lipid and carbohydrate side-chain modifications that regulate GPI-AP trafficking from the ER to the plasma membrane [51] (Fig. 1, Tables 1 and 2).

We compared the phenotypic abnormalities in patients with mutations in Synthesis and Transamidase+Remodeling Group genes. Several skeletal phenotypes were significantly more likely to occur in patients with mutations in the Synthesis stage of the biosynthetic pathway (Synthesis Group). The Synthesis Group had a greater occurrence (33% of patients) of *Abnormal digit morphology* (HPO terms listed using italics). Other phenotypes observed in the Synthesis Group patients were *Absent distal phalanges*, *Aplasia/Hypoplasia of fingers*, *Short digit*, *Broad finger* and *Broad toe*, *Clubbing*, *Clinodactyly*, and other abnormalities (Table 3). Transamidase+Remodeling Group patients (patients with mutations in the later stage genes) had fewer incidences of *Abnormal digit morphology* (6.7%, Table 3). For instance, Synthesis Group patients are statistically more likely to have *Short digit* (24% of the patients), whereas only one individual (< 2%) was indicated as having a *Short digit* in the Transamidase+Remodeling Group (Table 3).

**Table 3:** Significantly Overrepresented Synthesis-Group Phenotypes

HPO ID	Term Name	Synthesis Group (patients with phenotype/total)	Synthesis Group (%)	Transamidase + Remodeling Group (patients with phenotype/total)	Transamidase + Remodeling Group (%)	Corrected *p*-value
HP:0011297	Abnormality of digit	31/93	33.3	4/59	6.8	0.03185
HP:0011805	Abnormal muscle morphology	25/93	26.9	2/59	3.4	0.04678
HP:0011927	Short digit	22/93	23.7	1/59	1.7	0.04883
HP:0001367	Abnormal joint morphology	22/93	23.7	1/59	1.7	0.04883
HP:0100261	Abnormal tendon morphology	19/93	20.4	0/59	0.0	0.04346

**Fig. 2:**
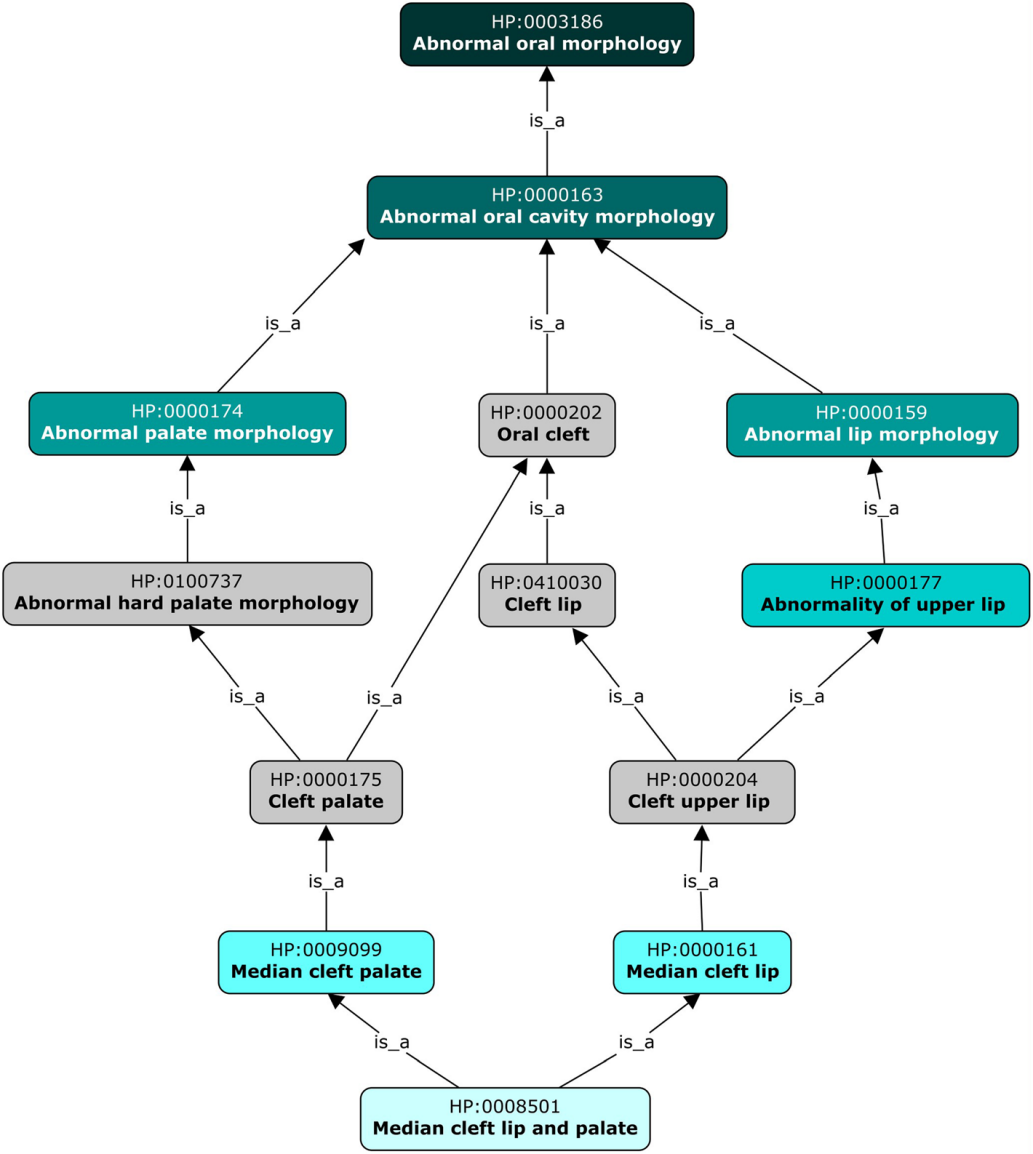
Example of HPO hierarchy. The hierarchy in the HPO for cleft palate and neighboring phenotypes

Moreover, Synthesis Group patients were significantly more likely to have *Abnormal muscle morphology, Abnormal tendon morphology,* and/or *Abnormal joint morphology*. This was mainly related to the term *Flexion contracture* or descendants thereof (Fig. 2). Eighteen of the ninety-three patients in the Synthesis group had a flexion contracture of one or more joints (descendant of *Flexion contracture*). A “contracture” is a shortening or hardening of the muscle or tendon that leads to the loss of motion of that joint and therefore is listed under the muscle, tendon, and joint hierarchies of the HPO. In addition to flexion contractures, a handful of other types of phenotypes add significance to these parent classes. One Synthesis Group patient [12] displayed *Abnormality of the Achilles tendon* which is a child of *Abnormal tendon morphology.* As for *Abnormality of joint morphology,* a single patient had *Axillary pterygia,* which is the presence of a cutaneous membrane in the armpit [31]. Additionally, several Synthesis Group patients and a single Transamidase+Remodeling Group patient had *Joint hypermobility* (Tables 1 and 2). Besides contractures, several other observed phenotypes contributed to the *Abnormal muscle morphology* phenotype being significantly increased in the Synthesis Group. Such phenotypes include *Muscle dystrophy*, *Camptodactyly*, *Generalized amyotrophy*, *Macroglossia*, *Myopathy*, *Rimmed vacuoles, Muscle fiber splitting*, *Skeletal muscle atrophy, Abnormal muscle, and fiber dystrophin expression*. Two Transamidase+Remodeling Group patients have noted *Abnormal muscle morphology*, but this group differs in the types of phenotypes reported (*Skeletal muscle atrophy* and *Increased muscle lipid content*).

Transamidase+Remodeling Group patients, overall, displayed more diverse phenotypic abnormalities that selectively affected this population as opposed to the Synthesis Group (Tables 3 and 4). The most common alterations were in bone and facial development and neurodevelopmental disabilities. The frequency of *Abnormality of bone density* is significantly higher in Transamidase+Remodeling Group patients as compared to Synthesis Group patients. *Osteopenia*, a reduction in bone mineral density below normal but not as severe as *Osteoporosis*, occurred in 22% of the Transamidase+Remodeling Group patients, while only 2% of the patients in the Synthesis Group were reported to have *Osteopenia*. *Osteopenia* contributes almost entirely to the significance identified in *Reduced bone mineral density (*parent term), *Abnormality of bone mineral density (*grandparent term), *Abnormal bone ossification* (great-grandparent term), and *Abnormal bone structure* (great-great-grandparent term) in the Transamidase+Remodeling Group patients. The only additional *Abnormal bone structure* phenotypes observed were a *Thin bony cortex* observed in a single patient in the Synthesis Group [52], and two patients were observed to have *Reduced bone mineral density and Osteoporosis* in one patient in the Transamidase+Remodeling Group [42] (Table 4).

**Table 4:** Significantly Overrepresented Transamidase+Remodeling-Group Phenotypes

HPO ID	Term Name	Synthesis Group (patients with phenotype/total)	Synthesis Group (%)	Transamidase+Remodeling Group (patients with phenotype/total)	Transamidase+Remodeling Group (%)	Corrected *p*-value
HP:0002360	Sleep disturbance	0/93	0.00	13/59	22.03	0.00047
HP:0012759	Neurodevelopmental abnormality	68/93	73.12	58/59	98.31	0.01235
HP:0000708	Behavioral abnormality	14/93	15.05	25/59	42.37	0.03610
HP:0012758	Neurodevelopmental delay	57/93	61.29	54/59	91.53	0.00898
HP:0001249	Intellectual disability	15/93	16.13	39/59	66.10	0.00000
HP:0003330	Abnormal bone structure	3/93	3.23	15/59	25.42	0.00773
HP:0011849	Abnormal bone ossification	2/93	2.15	15/59	25.42	0.00193
HP:0004348	Abnormality of bone mineral density	2/93	2.15	15/59	25.42	0.00193
HP:0004349	Reduced bone mineral density	2/93	2.15	15/59	25.42	0.00193
HP:0000938	Osteopenia	2/93	2.15	13/59	22.03	0.01305
HP:0000400	Macrotia	2/93	2.15	15/59	25.42	0.00193
HP:0002265	Large fleshy ears	1/93	1.08	14/59	23.73	0.00106
HP:0000366	Abnormality of the nose	26/93	27.96	36/59	61.02	0.01119
HP:0100737	Abnormal hard palate morphology	6/93	6.45	17/59	28.81	0.03743
HP:0000202	Oral cleft	6/93	6.45	18/59	30.51	0.01556
HP:0000175	Cleft palate	6/93	6.45	17/59	28.81	0.03743

Similarly, patients in the Transamidase+Remodeling Group, predominantly patients with *PGAP3* mutations, are significantly more likely to having *Macrotia*. The Transamidase+Remodeling Group patients were classified as having *Macrotia (large ears greater than 2x the standard deviation)* 25% of the time, while the incidence was only 2% for the Synthesis Group patients. The vast majority of these Transamidase+Remodeling Group patients were described as specifically having *Large fleshy ears*, a child of *Macrotia* (Table 4).

Other facial developmental abnormalities found in Transamidase+Remodeling Group patients were *Abnormal hard palate morphology* and its child term, *Cleft palate.* Both phenotypes occurred with significantly higher frequency in the Transamidase+Remodeling Group as compared to the Synthesis Group (29% vs. 6% of patients) (Table 4, Fig. 2). *Cleft palate* was the predominant phenotype identified 16 Transamidase+Remodeling Group patients (vs. 6 patients in the Synthesis Group), which resulted in both *Abnormal hard palate morphology* and *Cleft palate* reaching significance*.* Additionally, two patients in the Transamidase+Remodeling Group were described as having *Median cleft lip and palate,* great-grandchild of *Cleft palate,* which also contributed to the significance of these two phenotypes (Table 4, Fig. 2**)**. Because the term *Cleft palate* has multiple parents in the HPO, *Oral cleft* was also identified as selectively enriched in Transamidase+Remodeling Group patients. Besides the phenotypes already mentioned, the significance of the term *Oral cleft* stemmed from *Cleft upper lip* and *Cleft lip* (Table 4, Fig. 2).

Numerous patients in both groups have *Abnormality of the nose*, but patients in the Transamidase+Remodeling Group were significantly more likely to have alterations in their nose (Synthesis Group = 28% vs. Transamidase+Remodeling Group = 61%). Both groups have *Abnormalities of the nose*, such as *Broad nasal tip* and *Wide nasal bridge* being the most common in each group. While many of the nose abnormalities are present in both groups, *Prominent nose* was only found in the Transamidase+Remodeling Group (15%). *Prominent nose* appears to be strongly associated with mutations in *PGAP3* and was only reported in those patients. One patient in the Synthesis Group had a *Prominent nasal bridge* (Table 4).

There are numerous mental and cognitive phenotypes affecting both groups, however, the Remodeling Group appears to have been impacted more often. While both groups have a large percentage of patients with *Neurodevelopmental abnormality*, 98% of the Transamidase+Remodeling Group were noted with *Neurodevelopmental abnormality*, as opposed to 73% of the Synthesis Group. More specifically, the Transamidase+Remodeling Group had an increased incidence of *Neurodevelopmental delay*, *Intellectual disability*, and *Behavioral abnormality* (92, 66, and 42%, respectively) while the Synthesis Group had a significantly smaller population with these abnormalities (61, 16, and 15%, respectively) (Table 4).

The division we chose between Synthesis and Transamidase+Remodeling Groups is but one of many possible ways of dividing the GPI pathway, and we reasoned that other partitionings might display other phenotypic differences. In order to explore this, we defined a group consisting of the GPI synthesis genes as well as the transamidase complex genes (Synthesis+Transamidase Group) and compared it with the genes responsible for fatty-acid remodeling (Remodeling Group). The Remodeling group consists of the genes *PGAP1*, *PGAP3*, *PGAP2*, and *PGAP5* (a subset of the original Transamidase+Remodeling Group). The Synthesis+Transamidase Group showed enrichment of *Urinary tract anomalies*. The Remodeling group showed enrichment for some of the same terms as in the Transamidase+Remodeling Group, including *Behavioral abnormality, Neurodevelopmental delay, Abnormality of the hard palate, Oral cleft,* and *Cleft palate.* Additionally, the Remodelling group had *Decreased head circumference*, *Altered eye location*, *Ear* and *Eyelid morphology abnormalities*, *Wide nasal bridge*, *Upper lip abnormalities*, and *Elevated alkaline phosphatase* (Additional file 1: Table S3).

### Candidate causal genes for component phenotypes of the IGDs

Mutations in genes that encode enzymes of the GPI biosynthesis pathway result in mistargeting of GPI-APs [53], but the abnormal distribution of GPI-APs in the IGDs has not been characterized in detail. Our hypothesis is that mis-anchoring and, therefore, mistargeting of individual GPI-APs leads to dysfunction of the targeted proteins which in turn leads to some or all of the phenotypic abnormalities observed in the IGDs. A better understanding of the mistargeting of GPI-APs could, therefore, clarify the molecular pathogenesis of the IGDs and shed light on genotype-phenotype correlations.

Over 142 human proteins have been identified in UniProt as being GPI-anchored (Additional file 1: Table S2). Of these, 23 (or 16%) of these genes encoding for GPI-APs have been associated with at least one Mendelian disease (a total of 34 Mendelian diseases were identified), and therefore, numerous phenotypes that define these diseases. We did not observe a significant enrichment of Gene Ontology terms for the genes, nor an enrichment of Mammalian Phenotype Ontology terms (including embryonic lethality) among the orthologs of these genes (data not shown). Thirty-four phenotypes in patients with mutations in GPI-anchored genes overlap with the phenotypes of CDG patients (Table 1 and 2). The fact that GPI-biosynthesis gene mutation and GPI-anchored gene mutations may cause overlapping but not identical phenotypes is expected since mutations in the GPI-biosynthesis pathway would likely alter the activity and function of a number of GPI-anchored proteins, and therefore, multiple signaling pathways.

To further delve into the pathways affected by GPI-biosynthesis gene mutations, we investigated the phenotypes that were observed to be more frequent in the Synthesis or Transamidase+Remodeling Groups. In the Synthesis Group, genes associated with the 5 characteristic phenotypes (Table 3) were compared. A total of 102 genes were associated with Mendelian diseases that share each of the five phenotypic features (Additional file 1: Figure S1).

When comparing the genes associated with the Transamidase+Remodeling Group, two genes were associated with 15 of the 16 Transamidase+Remodeling Group enriched phenotypes: fibroblast growth factor receptor tyrosine kinase (*FGFR2*), and a downstream signaling partner, B-Raf (*BRAF*) (Additional file 1: Figure S2). FGFR2 and B-Raf are associated with all the Transamidase+Remodeling Group phenotypes except Large fleshy ears. Notably, these genes are associated with the parent term of *Large fleshy ears*, *Macrotia*. The exclusion of *Large fleshy ears* may be due to the fact that patients present with large ears but not *Large fleshy ears*, or it could be due to the specificity in which physicians are presenting patient data or the detail recorded by curators and researchers. Mutations in *FGFR2* are associated with over ten distinct diseases including Pfeiffer syndrome and Crouzon syndrome [16, 54]. Mutations in *BRAF* are associated with seven diseases including Noonan syndrome type 7 and Cardiofaciocutaneous syndrome.

**Fig. 3:**
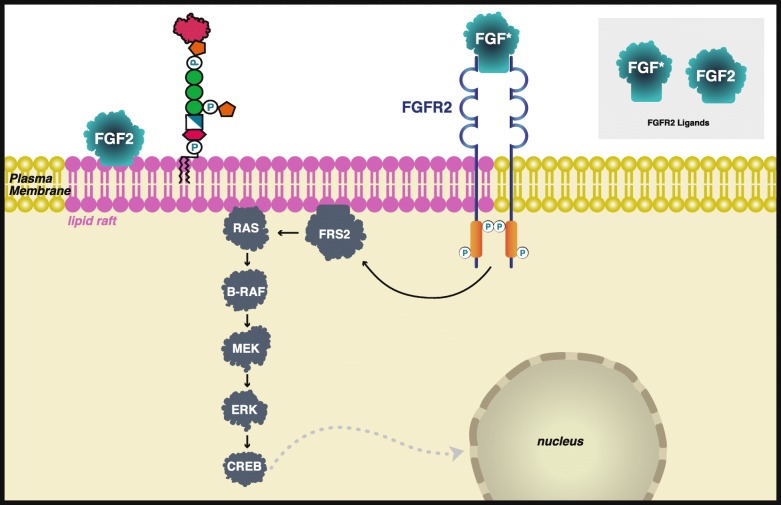
Schematic representation of FGFR2 signaling through the Ras/Raf/MAPK pathway. FGFR2 and B-Raf were found 2 associated with 15 of 16 phenotypes over-represented in the Transamidase+Remodeling Group and are in signaling cascades associated with lipid rafts which contain GPI-anchored proteins. *Numerous FGFs activate the FGFR2. Only FGF2 is known to be associated with lipid rafts (purple) [55–58]

Although neither FGFR2, a membrane-spanning protein, nor B-Raf has been identified as GPI-APs, FGFR2 has been shown to associate with lipid rafts in oligodendrocytes [55] and osteoblasts [56] and B-Raf translocation occurs more rapidly in the presence of lipid rafts [57]. GPI-APs are associated with lipid rafts [1, 7] suggesting that this may be a key altered pathway for Transamidase+Remodeling-Group-specific mutations (Fig. 3). Several other signaling partners within the FGFR2 pathway are also associated with lipid rafts, including ligand FGF2 [55, 58], and FRS2 [55]. There may be several targets or interactions with GPI-APs and FGFR2 signaling pathways.

Interestingly, two target proteins, GPC3 and GPC6 are associated with 25 of GPI-AP associated phenotypic abnormalities (Table 5). Both proteins are members of the glypican family of heparan sulfate proteoglycans that are bound to the cytoplasmic surface of the plasma membrane through a covalent GPI linkage. GPC3 can act as an FGFR1 and FGFR2 coreceptor required for the reception and subsequent relay of the FGF9 signals responsible for the control of coronary vascular development [59], suggesting a possible link.

**Table 5:** A selected list of GPI-anchored genes that when mutated give rise to overlapping phenotypes with CDG patients

HPO Term	GPI-anchored genes											
	APLI	ALPL	ALPP	ALPPL2	CNTN1	FOLR1	GPC6	GPC3	CD59	CNTN2	GPIHBP1	CD55
Hyperphosphatasia	x	x	x	x								
High palate					x							
Hypertelorism					x			x				
Muscular hypotonia					x			x	x			
Generalized hypotonia								x	x			
Polyhydramnios					x							
Feeding difficulties					x							
Camptodactyly					x							
Intellectual disability						x						
Seizures						x				x		
Malar flattening							x					
Epicanthus							x	x				
Micrognathia							x					
Depressed nasal bridge							x	x				
Wide nasal bridge							x	x				
Short neck							x					
Atrial septal defect							x					
Frontal bossing							x					
Short nose							x	x				
Tremor										x		
Cleft palate								x				
Coarse facial features								x				
Hepatomegaly								x			x	x
Macrocephaly								x				
Patent ductus arteriosus								x				
Short distal phalanx of finger								x				
Small nail								x				
Splenomegaly								x			x	
Anteverted nares								x				
Wide mouth								x				
Splenomegaly								x				
Failure to thrive											x	
Growth delay												x

## Discussion

The 29 GPI-biosynthesis enzymes are critical for building the GPI backbone, adding the GPI anchor to proteins, and targeting them to subcellular compartments. The first ~ 20 genes in the pathway are dedicated to building the GPI anchor, while the last ~ 9 genes anchor the proteins to the GPI backbone, and further modify the backbone, thereby regulating targeting of the anchored protein (Table 1). We hypothesized that the clinical features of diseases associated with either the Synthesis or the Transamidase+Remodeling stage of the GPI-biosynthesis pathway may be significantly distinct in each group. Although naively one might think that a mutation of any component of the GPI biosynthesis pathway would have identical phenotypic consequences, it is likely that defects in various components of the pathway affect the biochemistry and functions of GPI-anchored proteins in different ways. Our analysis showed 5 phenotypic abnormalities that were significantly more common with mutations in Synthesis pathway genes and 16 abnormalities that were more common with mutations in Transamidase+Remodeling pathway genes.

While our computational analysis is not able to identify the biochemical mechanisms underlying these differences, we can speculate about the range of factors that might be involved. IGDs do not lead to a uniform reduction of GPI-APs on all cells. For example, some patients with mental retardation, autosomal recessive 53 (MRT53) resulting from variants of PIGG have normal GPI expression on granulocytes while fibroblasts show a reduced global level of GPI anchors and of specific GPI-linked markers [60]. Therefore, one potential factor related to different phenotypic spectrums of the IGDs might be related to the distribution and degree of reduction of GPI-APs in different tissues [61].

The biochemical consequences of individual GPIs may be specific to particular tissues or developmental stages and would affect the target proteins, the GPI-APs, differentially, leading to different phenotypic consequences. The Synthesis Group displayed an enrichment of terms related to *Flexion contracture*, *Abnormal digit,* and *Short digit*, while the Synthesis Group showed an overrepresentation of *Neurodevelopmental abnormalities*, *Bone density abnormalities,* and additional facial developmental anomalies (Tables 3 and 4), which we speculate suggests a differential effect of mutations in Synthesis and Transamidase+Remodeling genes on the corresponding tissues.

The analyses of the Synthesis vs. Transamidase+Remodeling Groups, as well as the Synthesis+Transamidase vs. Remodelling groups, clearly demonstrate that the phenotypic abnormalities observed with mutations of genes involved in the GPI-biosynthesis pathway are not consistent across all of the genes. We limited our analysis to two comparisons and showed statistically significant differences in each case. As larger numbers of case reports become available, it may be possible to identify significant differences on a finer scale, involving smaller groups of genes, individual genes, or even specific variants. Understanding these differences may be helpful for precision management or even treatment of the GPI diseases in the future.

## Methods

### Patient selection

An extensive literature search was conducted using Clinvar [62], Pubmed, OMIM [63], and references for each GPI-anchoring gene to identify case studies. Searches were initiated using the terms “GPI”, “glycosylphosphatidylinositol”, and “mutation”, allowing citations in each publication to be looked up. Variants associated with each disease-related gene in the GPI pathway were examined in ClinVar to identify relevant case reports containing phenotypic descriptions. All case studies of individuals with pathogenic mutations in the GPI-anchoring synthesis pathway were included as long as they met the following criteria: an identifiable mutation was presented, phenotypic information about the patient was included, and the patient was not already included in another paper (See Tables 1, 2, Additional file 1: Table S4). Patient identifiers, phenotypes, and genetic variants were recorded.

### Biocuration

Biocuration was performed with an in-house and freely available Java desktop tool for the curation of case reports called HpoCaseAnnotator (https://github.com/monarch-initiative/HpoCaseAnnotator). The tool was used to enter and track PMID, variant information, patient ID, and phenotype(s) associated with each patient. HpoCaseAnnotator offers a concept recognition tool and other convenient functions to streamline HPO-based phenotype annotation. HpoCaseAnnotator calls VariantValidator [64] to check the HGVS syntax and chromosomal locations of variants.

### Analysis of the distribution of phenotypes in the selected groups

The biocurated case reports were used to analyze the distribution of phenotypic features within selected groups. Patients were classified into either Synthesis and Transamidase+Remodeling Groups or Synthesis+Transamidase and Remodeling Groups according to the gene mutation identified (Table 1**,** Additional file 1: Table S1, Additional file 1: Table S4). For each HPO term appearing in any proband record, the software counts the number of patients in each group annotated with that term. These counts are propagated upward in the HPO hierarchy so that a patient annotated with term *T* is included in the count for any term that subsumes *T* (i.e., for ancestors of *T* in the ontology). For instance, if a patient is annotated to *Flexion contracture of the 2nd finger* (HP:0009537), then implicitly the patient is also annotated to *Flexion contracture of finger* (HP:0012785) and *Abnormal 2nd finger morphology* (HP:0004100), as well as all the ancestor terms on the path to the root of the ontology. Using a *χ*^2^ test with one degree of freedom, the incidence of each phenotype term was compared between the two groups. Comparisons with insufficient data (expected value below 5 in any cell of the 2 × 2 contingency table) were omitted. A Bonferroni correction for multiple comparisons was applied to achieve α ≤ 0.05. The analysis was implemented as a Java application (code available at https://github.com/monarch-initiative/phenoCompare, release v1.0.0).

## Supplementary information

**Additional file 1: Figure S1.** Venn diagram representing overlaps of genes associated with the indicated phenotypic abnormalities in the Synthesis group. **Figure S2.** Venn diagram representing genes found in at diseases that overlap by at least 14 phenotypic abnormalities. **Table S1.** Case reports included in the present work. **Table S2.** GPI-anchored proteins. **Table S3.** Phenotypic comparison of Synthesis+Transamidase (S/T) vs. Remodeling groups. **Table S4.** Publications curated for this work.

## Data Availability

All data and materials are included in this published article and its supplementary information files. Information about the analysis tools can be found in the HpoCaseAnnotator Repository (https://github.com/monarch-initiative/HpoCaseAnnotator) or in phenoCompare Repository (https://github.com/monarch-initiative/phenoCompare).

## Abbreviations

BRAF: B-Raf
CDG: Congenital disorders of glycosylation
EIEE: Early Infantile Epileptic Encephalopathy
ER: Endoplasmic reticulum
EtNP: Phosphatidylinositol; ethanolamine phosphate
FGFR2: Fibroblast growth factor receptor tyrosine kinase
GlcN: D-Glucosamine
GlcNAc: N-Acetyl-D-glucosamine
GPI: Glycophosphatidylinositol
GPI-AP: GPI-anchored proteins
HPMRS: Hyperphosphatasia with mental retardation
HPO: Human phenotype ontology
IGD: Inherited GPI deficiencies
MRT53: Mental retardation, autosomal recessive 53
PGAP: Post-GPI attachment to proteins
PNH2: Paroxysmal nocturnal hemoglobinuria 2
