# Supplementary material for: Significantly different clinical phenotypes associated with mutations in synthesis and transamidase+remodeling glycosylphosphatidylinositol (GPI)-anchor biosynthesis genes
Source: Orphanet J Rare Dis. 2020 Feb 4;15:40. doi: 10.1186/s13023-020-1313-0 (PMC7001271; doi:10.1186/s13023-020-1313-0)
Supplement: Supplementary file 1 — Additional file 1: Figure S1. Venn diagram representing overlaps of genes associated with the indicated phenotypic abnormalities in the Synthesis group. Figure S2. Venn diagram representing genes found in at diseases that overlap by at least 14 phenotypic abnormalities. Table S1. Case reports included in the present work. Table S2. GPI-anchored proteins. Table S3. Phenotypic comparison of Synthesis+Transamidase (S/T) vs. Remodeling groups. Table S4. Publications curated for this work. [file 13023_2020_1313_MOESM1_ESM.pdf]

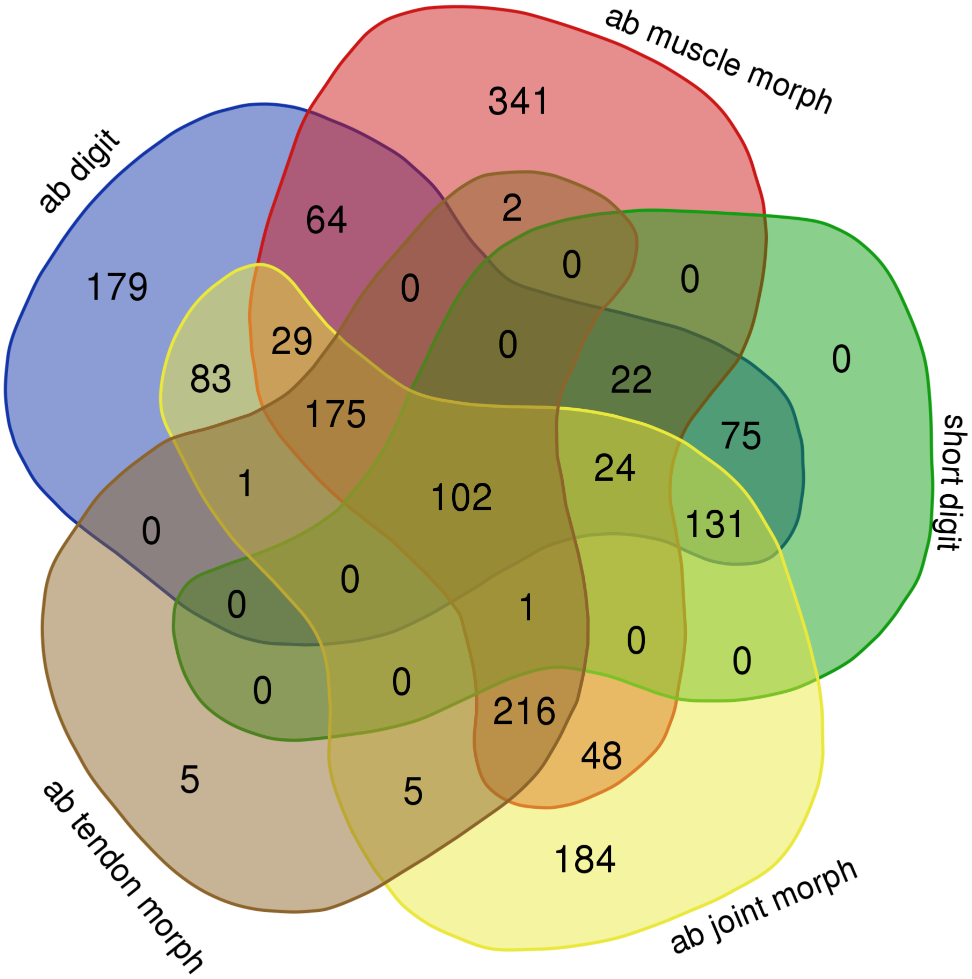

Figure **S1**: Venn diagram representing overlaps of genes associated with the indicated phenotypic abnormalities in the `Synthesis` group.

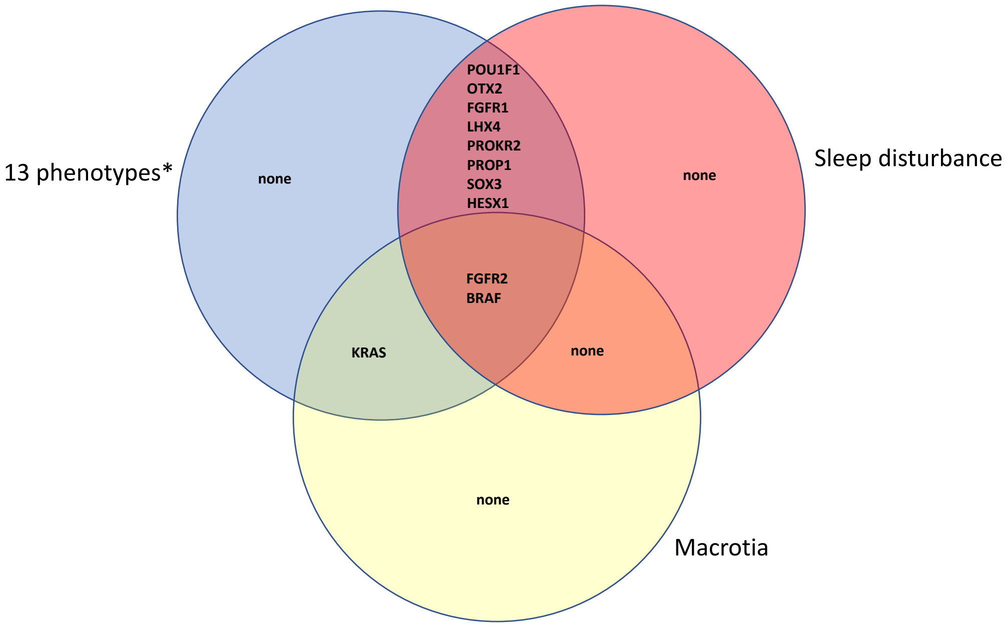

Figure **S2**: Venn diagram representing genes found in at diseases that overlap by at least 14 phenotypic abnormalities. Two genes, *FGFR2* and *BRAF*, were associated with Mendelian diseases that are characterized by all 15 phenotypic features. *KRAS* was associated with the 13 phenotypes as well as Macrotia. The 8 genes shown were listed with the 13 phenotypic features and Sleep disturbance. The 13 phenotypes were: Abnormal bone density, Abnormal bone ossification, Abnormal bone structure, Abnormality of nose, Behavioral abnormality, Cleft palate, Hard palate, Intellectual disability, Neurodevelopmental delay, Neurodevelopmental abnormality, Oral cleft, Osteopenia, Reduced bone density.

Table **S1**: Case reports included in the present work. The gene, patient id, and a list of the HPO terms that we curated for each patient are shown. S: Synthesis group. T+R: Transamidase+Remodeling group.

Gene	Patient ID	HPOs	Group	PMID
DPM1	PY	HP:0012448, HP:0004322, HP:0001643, HP:0100704, HP:0002015, HP:0002104, HP:0001250, HP:0000822, HP:0001272, HP:0000486, HP:0003186, HP:0005484, HP:0003236, HP:0007333	S	10642597
DPM1	CH	HP:0012448, HP:0006801, HP:0000174, HP:0002353, HP:0010844, HP:0002374, HP:0100704, HP:0001250, HP:0011398, HP:0001263, HP:0000961, HP:0005484, HP:0001028, HP:0005949, HP:0000494, HP:0003259, HP:0001009, HP:0000271	S	10642597
DPM1	Patient	HP:0001270, HP:0001250, HP:0011398, HP:0002457, HP:0005484, HP:0012385, HP:0009276, HP:0040081	S	23856421
DPM2	Patient 3	HP:0001999, HP:0002058, HP:0002650, HP:0002421, HP:0002197, HP:0011968, HP:0011947, HP:0040081, HP:0002803, HP:0030951, HP:0001250, HP:0011398, HP:0001263, HP:0003198, HP:0001321, HP:0001320, HP:0001344, HP:0005484	S	23109149
DPM2	Patient 2	HP:0001999, HP:0002058, HP:0002650, HP:0002421, HP:0002197, HP:0011968, HP:0011947, HP:0040081, HP:0002803, HP:0030951, HP:0000689, HP:0001250, HP:0000347, HP:0011398, HP:0001263, HP:0000486, HP:0003198, HP:0001321, HP:0001320, HP:0001344, HP:0005484	S	23109149
DPM2	Patient 1	HP:0002194, HP:0025404, HP:0002421, HP:0012157, HP:0002205, HP:0011947, HP:0000617, HP:0002910, HP:0000219, HP:0002705, HP:0006380, HP:0001250, HP:0000347, HP:0003196, HP:0011398, HP:0011344, HP:0001561, HP:0005484, HP:0100360, HP:0000294, HP:0008936, HP:0002240, HP:0002002, HP:0040246, HP:0002553, HP:0002098, HP:0030903, HP:0030235, HP:0002987, HP:0002878, HP:0000648, HP:0010460, HP:0100543, HP:0000601, HP:0000271, HP:0002090	S	23109149
DPM3	Patient	HP:0004302, HP:0003805, HP:0004322, HP:0003707, HP:0025502, HP:0001644, HP:0001763, HP:0003160, HP:0002401, HP:0002910, HP:0002515, HP:0003487, HP:0005109, HP:0003560, HP:0003198, HP:0003701, HP:0009053, HP:0001928, HP:0003259, HP:0003555, HP:0001315	S	19576565
DPM3	Patient	HP:0004302, HP:0003805, HP:0004322, HP:0003707, HP:0025502, HP:0001644, HP:0001763, HP:0003160, HP:0002401, HP:0002910, HP:0002515, HP:0003487, HP:0005109, HP:0003560, HP:0003198, HP:0003701, HP:0009053, HP:0001928, HP:0003259, HP:0003555, HP:0001315	S	19576565

Continued on next page

Table **S1** – *Continued from previous page*

Gene	Patient ID	HPOs	Group	PMID
MPDU1	Patient S	HP:0040189, HP:0001250, HP:0008064, HP:0003510, HP:0011344, HP:0001541, HP:0001276, HP:0011968, HP:0001510, HP:0012050, HP:0040189, HP:0040196, HP:0001698, HP:0001873, HP:0011968, HP:0002119, HP:0002521, HP:0000803, HP:0000648, HP:0012050, HP:0001250, HP:0003510, HP:0008064, HP:0011344, HP:0001541, HP:0001276, HP:0001510, HP:0012704, HP:0040189, HP:0001319, HP:0011968, HP:0012050, HP:0001250, HP:0003510, HP:0008064, HP:0011344, HP:0001541, HP:0000233, HP:0001276, HP:0000242, HP:0001510, HP:0000260	S	11733564
PIGA	III-7 Family B	HP:0012469, HP:0001263, HP:0002521	S	24357517
PIGA	III-13 Family B	HP:0001250, HP:0001263, HP:0002521, HP:0011947	S	24357517
PIGA	III-11 Family B	HP:0002133, HP:0002069, HP:0001263, HP:0002521, HP:0000969	S	24357517
PIGA	III-8 Family B	HP:0012469, HP:0001272, HP:0001263, HP:0002521, HP:0002500, HP:0002090	S	24357517
PIGA	IV-2 Family B	HP:0001270, HP:0008936, HP:0001250, HP:0001263, HP:0002521, HP:0001336	S	24357517
PIGA	IV-4	HP:0002079, HP:0002123, HP:0001623, HP:0001643, HP:0002878, HP:0002139, HP:0003155, HP:0002705, HP:0001371, HP:0001252, HP:0001561, HP:0001321, HP:0025116, HP:0011330, HP:0001414, HP:0001348	S	22305531
PIGA	IV-2	HP:0002079, HP:0006956, HP:0005280, HP:0001623, HP:0000076, HP:0001631, HP:0001792, HP:0002104, HP:0002714, HP:0006380, HP:0003273, HP:0000212, HP:0001321, HP:0001331, HP:0009381, HP:0001169, HP:0000272, HP:0000470, HP:0000160, HP:0002123, HP:0002987, HP:0000239, HP:0000269, HP:0000201, HP:0000463, HP:0000396, HP:0000582, HP:0001348, HP:0002090, HP:0000081	S	22305531
PIGA	IV-4	HP:0002123, HP:0002079, HP:0001250, HP:0001371, HP:0010851, HP:0011398, HP:0000076, HP:0001347, HP:0012430, HP:0000271, HP:0003155, HP:0002123, HP:0002079, HP:0001371, HP:0010851, HP:0011398, HP:0001561, HP:0001367, HP:0001347, HP:0012430, HP:0000271	S	24706016
PIGA	5	HP:0002187, HP:0003155, HP:0002079, HP:0011398, HP:0002521, HP:0001347, HP:0002445, HP:0000271	S	24706016
PIGA	4	HP:0003155, HP:0002079, HP:0001250, HP:0001371, HP:0002342, HP:0001367, HP:0002510, HP:0002521, HP:0003429, HP:0000271, HP:0000717	S	24706016
PIGA	3	HP:0002187, HP:0002197, HP:0010818, HP:0000717	S	24706016
PIGA	2	HP:0002187, HP:0003155, HP:0002123, HP:0002079, HP:0001250, HP:0001367, HP:0002521, HP:0002510, HP:0001336, HP:0012430, HP:0000271	S	24706016
PIGA	1	HP:0002187, HP:0002079, HP:0010851, HP:0001371, HP:0011398, HP:0000076, HP:0001561, HP:0005692, HP:0012430, HP:0002445, HP:0000271, HP:0010818	S	24706016
PIGA	IV-2	HP:0002123, HP:0002079, HP:0001371, HP:0001250, HP:0010851, HP:0011398, HP:0000076, HP:0001347, HP:0012430, HP:0000271	S	24706016

Continued on next page

Gene	Patient ID	HPOs	Group	PMID
PIGA	IV-3	HP:0001939, HP:0003121, HP:0001433, HP:0003487, HP:0003700, HP:0001257, HP:0001397, HP:0000365, HP:0002078, HP:0000496, HP:0002240, HP:0000684, HP:0001298, HP:0001251, HP:0000248, HP:0001332, HP:0003281, HP:0100704, HP:0001250, HP:0011398, HP:0040130	S	24259288
PIGA	III-10	HP:0040134, HP:0001712, HP:0001744, HP:0002174, HP:0012103, HP:0001873, HP:0001760, HP:0007190, HP:0200034, HP:0009909, HP:0002529, HP:0001250, HP:0001371, HP:0011952, HP:0000684, HP:0001541, HP:0001298, HP:0005484, HP:0001399, HP:0000496, HP:0001939, HP:0001357, HP:0002240, HP:0002133, HP:0006349, HP:0004322, HP:0002650, HP:0100704, HP:0002753, HP:0002119, HP:0002599, HP:0000029, HP:0003202, HP:0000687, HP:0001257, HP:0000691, HP:0003765, HP:0000980, HP:0001413, HP:0002171	S	24259288
PIGA	III-9	HP:0002133, HP:0008936, HP:0100704, HP:0002120, HP:0002540, HP:0030903, HP:0001182, HP:0011951, HP:0001250, HP:0000365, HP:0001344, HP:0005484, HP:0001433, HP:0001336, HP:0009085, HP:0001348, HP:0000280	S	24259288
PIGA	II:2	HP:0002123, HP:0001520, HP:0012736, HP:0012385, HP:0001371, HP:0002236, HP:0006191, HP:0005484, HP:0002063, HP:0012745, HP:0000324, HP:0012469, HP:0000637, HP:0008936, HP:0004673, HP:0005989, HP:0000248, HP:0002705, HP:0000316, HP:0002267, HP:0002521, HP:0001250, HP:0000474, HP:0001357, HP:0005469, HP:0000311, HP:0000337, HP:0100538, HP:0002056, HP:0001488, HP:0002835, HP:0000545, HP:0004308, HP:0002059, HP:0000365, HP:0000126, HP:0004446, HP:0006994, HP:0002104, HP:0002240, HP:0001667, HP:0000543, HP:0003155, HP:0007370, HP:0030515, HP:0000556, HP:0001103, HP:0031145, HP:0031145	S	25885527
PIGA	III-1	HP:0009890, HP:0002079, HP:0002271, HP:0002376, HP:0001631, HP:0001695, HP:0001671, HP:0002059, HP:0002104, HP:0001250, HP:0000674, HP:0001561, HP:0000288, HP:0012704, HP:0012429, HP:0002373, HP:0008936, HP:0002266, HP:0002795, HP:0002197, HP:0002119, HP:0003270, HP:0003155, HP:0001263, HP:0200134, HP:0000233, HP:0009085, HP:0000582	S	24259184
PIGA	Older twin	HP:0002133, HP:0005280, HP:0002123, HP:0002179, HP:0002069, HP:0002421, HP:0025404, HP:0002376, HP:0012001, HP:0000817, HP:0000316, HP:0001250, HP:0011168, HP:0001252, HP:0000463	S	29502866
PIGA	Younger twin	HP:0002133, HP:0005280, HP:0002179, HP:0002069, HP:0002421, HP:0025404, HP:0002376, HP:0012001, HP:0000817, HP:0000316, HP:0001250, HP:0001252, HP:0000463	S	29502866
PIGC	FamilyA II-4	HP:0001270, HP:0002069, HP:0001250, HP:0001263, HP:0010864, HP:0000750, HP:0031491	S	27694521
PIGC	FamilyA II-2	HP:0001270, HP:0002069, HP:0001250, HP:0001263, HP:0010864, HP:0000750, HP:0031491, HP:0100702	S	27694521
PIGC	FamilyB II-1	HP:0001250, HP:0001263, HP:0001249	S	27694521

Continued on next page

Table **S1** – *Continued from previous page*

Gene	Patient ID	HPOs	Group	PMID
PIGG	JP01	HP:0001250, HP:0004396, HP:0001263, HP:0000750, HP:0001252, HP:0001321, HP:0001510, HP:0002059, HP:0000717	S	26996948
PIGG	PK01	HP:0001263, HP:0001250, HP:0006829, HP:0007370, HP:0011343, HP:0001344, HP:0000729, HP:0008855, HP:0030047	S	26996948
PIGG	PK02	HP:0001263, HP:0001250, HP:0006829, HP:0007370, HP:0011343, HP:0001344, HP:0000729, HP:0008855, HP:0030047	S	26996948
PIGG	EG01	HP:0001250, HP:0001382, HP:0011398, HP:0011343, HP:0001263, HP:0006829, HP:0007370, HP:0000729, HP:0001344, HP:0008855, HP:0030047	S	26996948
PIGG	EG02	HP:0001250, HP:0001382, HP:0011343, HP:0001263, HP:0006829, HP:0007370, HP:0000729, HP:0001344, HP:0008855, HP:0030047	S	26996948
PIGH	Proband	HP:0002373, HP:0045045, HP:0002311, HP:0012343, HP:0000717, HP:0001290, HP:0000218, HP:0011343, HP:0000750, HP:0010529, HP:0001007	S	29603516
PIGL	Patient 10	HP:0100806, HP:0002098, HP:0000126, HP:0002644, HP:0002136, HP:0000316, HP:0003155, HP:0001250, HP:0001263, HP:0001156, HP:0000972, HP:0008070, HP:0000396, HP:0007503, HP:0000480, HP:0001249	S	29473937
PIGL	girl	HP:0006956, HP:0005280, HP:0002194, HP:0002421, HP:0002750, HP:0010546, HP:0004042, HP:0001792, HP:0011927, HP:0000637, HP:0000218, HP:0001250, HP:0011398, HP:0000684, HP:0000486, HP:0000365, HP:0010804, HP:0000280, HP:0000158, HP:0002789, HP:0000316, HP:0003155, HP:0011800, HP:0001320, HP:0000364, HP:0000463, HP:0001344, HP:0000293, HP:0006956, HP:0005280, HP:0002194, HP:0002421, HP:0002750, HP:0010546, HP:0004042, HP:0001792, HP:0011927, HP:0000637, HP:0000218, HP:0001250, HP:0011398, HP:0000684, HP:0000486, HP:0000365, HP:0010804, HP:0000280, HP:0000158, HP:0002789, HP:0000316, HP:0003155, HP:0011800, HP:0001320, HP:0000364, HP:0000463, HP:0001344, HP:0000293	S	25706356
PIGL	3988	HP:0400005, HP:0010783, HP:0005280, HP:0004209, HP:0001682, HP:0000126, HP:0000989, HP:0002136, HP:0011927, HP:0002007, HP:0000405, HP:0000589, HP:0001250, HP:0000369, HP:0000358, HP:0008064, HP:0000343, HP:0000271, HP:0001019	S	22444671
PIGL	0680-2-3	HP:0000175, HP:0002286, HP:0002213, HP:0012471, HP:0000316, HP:0000589, HP:0001250, HP:0000248, HP:0008064, HP:0011069, HP:0000687, HP:0000972, HP:0000431, HP:0001288, HP:0008591, HP:0008070, HP:0000396, HP:0000286, HP:0100040, HP:0001249, HP:0001638	S	22444671
PIGL	0680-2-2	HP:0000175, HP:0002286, HP:0002213, HP:0002059, HP:0012471, HP:0000316, HP:0000457, HP:0000589, HP:0001250, HP:0000248, HP:0008064, HP:0011069, HP:0000687, HP:0000972, HP:0000431, HP:0001288, HP:0008591, HP:0008070, HP:0000396, HP:0000286, HP:0100040, HP:0001249, HP:0001638	S	22444671

Continued on next page

Gene	Patient ID	HPOs	Group	PMID
PIGL	33300	HP:0010047, HP:0000193, HP:0005280, HP:0010049, HP:0100759, HP:0025474, HP:0002213, HP:0002059, HP:0000958, HP:0100542, HP:0100760, HP:0001270, HP:0000589, HP:0008064, HP:0000894, HP:0000377, HP:0008070, HP:0009931, HP:0000286, HP:0000252, HP:0001169, HP:0001999, HP:0001769, HP:0001945, HP:0010783, HP:0004279, HP:0001943, HP:0010038, HP:0012211, HP:0000989, HP:0002901, HP:0012471, HP:0030148, HP:0005469, HP:0000405, HP:0000316, HP:0000455, HP:0007808, HP:0001156, HP:0000322, HP:0000972, HP:0000463, HP:0001598, HP:0100040, HP:0001805	S	22444671
PIGL	0682-2-1	HP:0002286, HP:0002213, HP:0002059, HP:0012471, HP:0000405, HP:0000316, HP:0000589, HP:0001250, HP:0000248, HP:0008064, HP:0011069, HP:0000687, HP:0000972, HP:0000431, HP:0001288, HP:0008070, HP:0000396, HP:0000286, HP:0100040, HP:0001249, HP:0001638	S	22444671
PIGL	277013	HP:0002069, HP:0002194, HP:0000154, HP:0030084, HP:0006347, HP:0010055, HP:0000107, HP:0000958, HP:0003155, HP:0000405, HP:0000965, HP:0000767, HP:0001263, HP:0000687, HP:0000365, HP:0009381, HP:0003764	S	28327575
PIGM	Family 2-2B	HP:0002121, HP:0030242, HP:0030243	S	16767100
PIGM	Family 2-2C	HP:0002121, HP:0030242, HP:0030243	S	16767100
PIGM	Family 1-1B	HP:0002121, HP:0030242, HP:0030243	S	16767100
PIGN	Patient	HP:0001626, HP:0002089, HP:0001831, HP:0030030, HP:0000054, HP:0000175, HP:0000110, HP:0012165, HP:0002623, HP:0002566, HP:0000028, HP:0001060, HP:0025193, HP:0003244, HP:0000776, HP:0000316, HP:0000445, HP:0000377, HP:0000463, HP:0000271	S	24852103
PIGN	COLL-2.3	HP:0000174, HP:0000110, HP:0000803, HP:0012718	S	27038415
PIGN	PIGN;V-2	HP:0001615, HP:0002100, HP:0000072, HP:0001631, HP:0000034, HP:0000639, HP:0001290, HP:0000219, HP:0000646, HP:0002705, HP:0000400, HP:0000278, HP:0000486, HP:0000341, HP:0001347, HP:0001337, HP:0011271, HP:0001945, HP:0000194, HP:0002020, HP:0002286, HP:0000126, HP:0000319, HP:0002305, HP:0010880, HP:0000316, HP:0001182, HP:0000269, HP:0001263, HP:0002090	S	21493957
PIGN	V-1	HP:0004467, HP:0000194, HP:0001655, HP:0000034, HP:0004969, HP:0000639, HP:0000646, HP:0002705, HP:0000347, HP:0000269, HP:0001156, HP:0009748, HP:0000774, HP:0000341, HP:0010804, HP:0000280	S	21493957
PIGN	NSGC-7.4	HP:0000174, HP:0000110, HP:0000803, HP:0012718	S	27038415
PIGN	Patient 1	HP:0002384, HP:0012448, HP:0000076, HP:0010544, HP:0000010, HP:0000639, HP:0012381, HP:0002705, HP:0001272, HP:0000347, HP:0011398, HP:0000341, HP:0003700, HP:0000286, HP:0010804, HP:0003324, HP:0001337, HP:0000194, HP:0004396, HP:0002119, HP:0000269, HP:0001263, HP:0001344, HP:0010506	S	24253414

Continued on next page

Gene	Patient ID	HPOs	Group	PMID
PIGN	Patient2	HP:0002384, HP:0012448, HP:0000076, HP:0010544, HP:0000010, HP:0000639, HP:0012381, HP:0002705, HP:0001272, HP:0000347, HP:0011398, HP:0000341, HP:0003700, HP:0000286, HP:0010804, HP:0003324, HP:0001337, HP:0000194, HP:0004396, HP:0002119, HP:0000269, HP:0001263, HP:0001344, HP:0010506	S	24253414
PIGN	male	HP:0002384, HP:0012703, HP:0000174, HP:0002194, HP:0002421, HP:0002353, HP:0002457, HP:0002533, HP:0004305, HP:0003196, HP:0001263, HP:0100370, HP:0100702, HP:0001344, HP:0100371	S	29096607
PIGN	NSGC-7.3	HP:0000174, HP:0000110, HP:0000803, HP:0012718	S	27038415
PIGN	Patient 2	HP:0006855, HP:0006956, HP:0100660, HP:0002123, HP:0001272, HP:0025439, HP:0001250, HP:0010544, HP:0000010, HP:0001336, HP:0001337, HP:0001290	S	26879448
PIGN	259633	HP:0100543, HP:0001250, HP:0012444, HP:0002194, HP:0007308, HP:0002205, HP:0001344	S	28327575
PIGO	II-1 family A	HP:0003155, HP:0001252, HP:0001263, HP:0010804, HP:0002025, HP:0000316, HP:0000750, HP:0001270, HP:0004322, HP:0000455, HP:0000431, HP:0006118, HP:0000076, HP:0000637, HP:0002251	S	22683086
PIGO	II-2 family A	HP:0003155, HP:0001252, HP:0001263, HP:0010804, HP:0002025, HP:0000316, HP:0004322, HP:0000750, HP:0001270, HP:0000455, HP:0000431, HP:0006118, HP:0000076, HP:0000637	S	22683086
PIGO	II-1 family B	HP:0001250, HP:0002265, HP:0001263, HP:0011316, HP:0004969, HP:0000324, HP:0010804, HP:0000316, HP:0002540, HP:0001270, HP:0000637, HP:0000494, HP:0000508, HP:0001792, HP:0003155, HP:0004209, HP:0000455, HP:0009642, HP:0000431, HP:0001631, HP:0006118, HP:0001800, HP:0000252, HP:0009909	S	22683086
PIGO	Boy	HP:0009882, HP:0002251, HP:0001636, HP:0002134, HP:0008527, HP:0012501, HP:0002871, HP:0002352, HP:0002104, HP:0003155, HP:0001272, HP:0011398, HP:0000365, HP:0000297, HP:0001344, HP:0009381, HP:0003429, HP:0031165, HP:0000271	S	24049131
PIGO	Individual II-1	HP:0002384, HP:0002069, HP:0002063, HP:0010864, HP:0002510, HP:0002059, HP:0003155, HP:0002705, HP:0001250, HP:0001263, HP:0200134, HP:0001252, HP:0002072, HP:0010804, HP:0100275	S	24417746
PIGO	Individual II-2	HP:0002069, HP:0001263	S	24417746
PIGO	263039	HP:0002251, HP:0002013, HP:0001810, HP:0002164, HP:0000407, HP:0000219, HP:0001270, HP:0003155, HP:0001263, HP:0001156, HP:0000364, HP:0001344, HP:0000252, HP:0001817, HP:0001804	S	28327575
PIGO	Patient 1	HP:0002384, HP:0002100, HP:0008936, HP:0002079, HP:0002120, HP:0011968, HP:0000817, HP:0002307, HP:0004305, HP:0000758, HP:0008763, HP:0002835, HP:0000711, HP:0001263, HP:0003560, HP:0001298, HP:0002093, HP:0002090	S	28900819
PIGO	Patient 2	HP:0012448, HP:0002100, HP:0008936, HP:0002079, HP:0012736, HP:0001250, HP:0001263, HP:0002353, HP:0000961, HP:0002120, HP:0002307, HP:0010818	S	28900819

Continued on next page

Table **S1** – *Continued from previous page*

Gene	Patient ID	HPOs	Group	PMID
PIGP	Male proband	HP:0002187, HP:0002079, HP:0002353, HP:0010546, HP:0002457, HP:0006895, HP:0002835, HP:0006380, HP:0003037, HP:0001250, HP:0011398, HP:0000961, HP:0001344, HP:0001357, HP:0002094, HP:0001618, HP:0200049	S	28334793
PIGP	Female proband	HP:0002100, HP:0010553, HP:0001250, HP:0002353, HP:0100704, HP:0000817, HP:0001347, HP:0002521, HP:0002169, HP:0002835, HP:0000549	S	28334793
PIGQ	Patient	HP:0010851, HP:0001371, HP:0012443, HP:0001250	S	24463883
PIGV	Patient 5	HP:0000750, HP:0011344, HP:0001792, HP:0009381, HP:0003155, HP:0010804, HP:0000283, HP:0009882, HP:0001831, HP:0000271, HP:0001336	S	24129430
PIGV	Patient 8	HP:0002251, HP:0000072, HP:0002025, HP:0006118, HP:0000076, HP:0000021, HP:0001792, HP:0000126, HP:0010055, HP:0003155, HP:0001250, HP:0011344, HP:0000750, HP:0000431, HP:0001344, HP:0009381, HP:0010804, HP:0000271	S	24129430
PIGV	Patient 9	HP:0001804, HP:0000455, HP:0001821, HP:0003155, HP:0000316, HP:0000126, HP:0200007, HP:0010804, HP:0000431, HP:0000175, HP:0001629, HP:0000072	S	24129430
PIGV	Patient 1	HP:0040255, HP:0002023, HP:0002251, HP:0000175, HP:0005807, HP:0002335, HP:0000126, HP:0002334, HP:0003155, HP:0003037, HP:0000316, HP:0000455, HP:0009748, HP:0009617, HP:0005781, HP:0001629	S	24129430
PIGV	Patient 7	HP:0004378, HP:0002251, HP:0002194, HP:0001655, HP:0000175, HP:0000126, HP:0001195, HP:0003155, HP:0001250, HP:0000455, HP:0001252, HP:0011344, HP:0000750, HP:0009836, HP:0001344, HP:0005484, HP:0000271	S	24129430
PIGV	Patient 2	HP:0009882, HP:0002251, HP:0001821, HP:0003155, HP:0000316, HP:0000455, HP:0001252, HP:0011344, HP:0000750, HP:0009836, HP:0000431, HP:0005484, HP:0010804, HP:0000271, HP:0010819	S	24129430
PIGV	Patient 3	HP:0002251, HP:0006118, HP:0000077, HP:0003155, HP:0000316, HP:0001250, HP:0000455, HP:0011398, HP:0001252, HP:0011344, HP:0000750, HP:0009836, HP:0000431, HP:0010804, HP:0000271	S	24129430
PIGV	Patient 6	HP:0003155, HP:0000316, HP:0002251, HP:0002194, HP:0000455, HP:0011398, HP:0001263, HP:0000750, HP:0006895, HP:0000271, HP:0000081, HP:0002814	S	24129430
PIGV	Patient 4	HP:0002123, HP:0005490, HP:0001792, HP:0003155, HP:0000316, HP:0000767, HP:0001250, HP:0000455, HP:0001252, HP:0011344, HP:0000750, HP:0000431, HP:0009381, HP:0005914, HP:0010804, HP:0000271	S	24129430
PIGV	Patient 1	HP:0006118, HP:0002069, HP:0002019, HP:0001792, HP:0000637, HP:0002714, HP:0000426, HP:0001270, HP:0011398, HP:0000311, HP:0000256, HP:0010804, HP:0001545, HP:0009894, HP:0000391, HP:0004378, HP:0002650, HP:0000126, HP:0003155, HP:0011343, HP:0000378, HP:0001520, HP:0001510, HP:0001344, HP:0000271, HP:0010819	S	22315194

Continued on next page

Table **S1** – *Continued from previous page*

Gene	Patient ID	HPOs	Group	PMID
PIGV	Patient 3	HP:0000072, HP:0006118, HP:0002883, HP:0001195, HP:0001250, HP:0000303, HP:0011398, HP:0011344, HP:0010804, HP:0100023, HP:0200006, HP:0000280, HP:0004378, HP:0010864, HP:0000077, HP:0000126, HP:0005326, HP:0000316, HP:0000699, HP:0003155, HP:0001061, HP:0011343, HP:0001263, HP:0000750, HP:0001562, HP:0010746, HP:0001510	S	22315194
PIGV	Patient 2	HP:0002251, HP:0002013, HP:0001623, HP:0002558, HP:0002714, HP:0000218, HP:0001270, HP:0001250, HP:0000256, HP:0005484, HP:0010804, HP:0009894, HP:0000391, HP:0004378, HP:0040262, HP:0012374, HP:0003270, HP:0000126, HP:0003155, HP:0001263, HP:0000378, HP:0010946, HP:0001520, HP:0001510, HP:0001344, HP:0000271	S	22315194
PIGV	Patient 4	HP:0003155, HP:0000316, HP:0001250, HP:0002069, HP:0011398, HP:0001263, HP:0000431, HP:0001344, HP:0010804, HP:0001525, HP:0000280	S	22315194
PIGW	Patient	HP:0001999, HP:0003155, HP:0000431, HP:0000023, HP:0012310, HP:0002521, HP:0010804	S	24367057
PIGW	Patient 1	HP:0001270, HP:0001250, HP:0001252, HP:0002098, HP:0001662, HP:0002527, HP:0010296, HP:0010819	S	27626616
PIGW	Patient 2	HP:0002607, HP:0001270, HP:0002123, HP:0001250, HP:0001263, HP:0001252, HP:0000158, HP:0010819	S	27626616
PIGY	Family A-II-1	HP:0006965, HP:0006528, HP:0002013, HP:0004387, HP:0006118, HP:0004691, HP:0002376, HP:0000938, HP:0011947, HP:0000518, HP:0002987, HP:0011927, HP:0025152, HP:0003155, HP:0006380, HP:0001250, HP:0001385, HP:0001510, HP:0031165, HP:0003236	S	26293662
PIGY	Family B-II-2	HP:0000504, HP:0001263, HP:0000750, HP:0000486, HP:0005484, HP:0000718	S	26293662
PIGY	Family B-II-3	HP:0002194, HP:0000752, HP:0001263, HP:0000750, HP:0008050, HP:0000252, HP:0000271, HP:0000154	S	26293662
PIGY	Family A-II-2	HP:0000023, HP:0000938, HP:0010943, HP:0002027, HP:0011927, HP:0000519, HP:0002835, HP:0003155, HP:0002617, HP:0001250, HP:0003273, HP:0001561, HP:0009826, HP:0001510, HP:0003236	S	26293662
GPAA1	1a	HP:0002066, HP:0002123, HP:0001272, HP:0001263, HP:0001310, HP:0000938, HP:0025190, HP:0000639, HP:0001249, HP:0011170, HP:0010819	T+R	29100095
GPAA1	1b	HP:0002066, HP:0001260, HP:0001272, HP:0001263, HP:0001310, HP:0000938, HP:0025190, HP:0000639, HP:0001249, HP:0010819	T+R	29100095
GPAA1	3a	HP:0004322, HP:0002069, HP:0002464, HP:0000938, HP:0000639, HP:0001290, HP:0000316, HP:0001272, HP:0000411, HP:0001263, HP:0000520, HP:0000341, HP:0000252, HP:0001249	T+R	29100095
GPAA1	3b	HP:0002123, HP:0001272, HP:0002069, HP:0000411, HP:0001263, HP:0000445, HP:0000520, HP:0000938, HP:0000639, HP:0001249, HP:0001290	T+R	29100095
GPAA1	4a	HP:0002066, HP:0004322, HP:0000938, HP:0000639, HP:0001272, HP:0001263, HP:0001257, HP:0000463, HP:0000341, HP:0011220, HP:0001249	T+R	29100095
GPAA1	4b	HP:0002066, HP:0004322, HP:0000938, HP:0001272, HP:0001263, HP:0001310, HP:0001257, HP:0000463, HP:0000341, HP:0011220, HP:0001249	T+R	29100095

Continued on next page

Table **S1** – *Continued from previous page*

Gene	Patient ID	HPOs	Group	PMID
GPAA1	4c	HP:0002066, HP:0004322, HP:0000938, HP:0001260, HP:0001272, HP:0001263, HP:0001310, HP:0001257, HP:0000463, HP:0011220, HP:0001249	T+R	29100095
GPAA1	2	HP:0002069, HP:0001263, HP:0100704, HP:0000938, HP:0000463, HP:0000341, HP:0011220, HP:0001249, HP:0001290	T+R	29100095
GPAA1	5b	HP:0002066, HP:0001260, HP:0002069, HP:0001272, HP:0001263, HP:0000445, HP:0001310, HP:0001249, HP:0001290	T+R	29100095
GPAA1	5a	HP:0002066, HP:0001260, HP:0002121, HP:0002069, HP:0001272, HP:0001263, HP:0001310, HP:0001249, HP:0010819, HP:0001290	T+R	29100095
PGAP1	Proband	HP:0002099, HP:0001319, HP:0001263, HP:0000486, HP:0009748, HP:0009904, HP:0011968, HP:0000395, HP:0000582, HP:0000639, HP:0000490, HP:0002099, HP:0001319, HP:0001263, HP:0000486, HP:0009748, HP:0009904, HP:0011968, HP:0000395, HP:0000582, HP:0000639, HP:0000490	T+R	25804403
PGAP1	1.2	HP:0000733, HP:0008936, HP:0001270, HP:0004322, HP:0000556, HP:0002791, HP:0000248, HP:0001263, HP:0011344, HP:0000750, HP:0009062, HP:0002090	T+R	26050939
PGAP1	1.1	HP:0000193, HP:0004322, HP:0006934, HP:0001508, HP:0001642, HP:0001631, HP:0010055, HP:0000504, HP:0000316, HP:0000556, HP:0000767, HP:0001263, HP:0001252, HP:0000750, HP:0000664, HP:0001276, HP:0001344, HP:0005484	T+R	26050939
PGAP1	II-1	HP:0006801, HP:0012444, HP:0002421, HP:0002098, HP:0000639, HP:0002104, HP:0001290, HP:0001270, HP:0003487, HP:0000750, HP:0025047, HP:0011400, HP:0005484, HP:0000496, HP:0001336, HP:0001337	T+R	27206732
PGAP1	II-2	HP:0012547, HP:0006801, HP:0012444, HP:0002421, HP:0002098, HP:0012101, HP:0000639, HP:0002104, HP:0001290, HP:0030874, HP:0001270, HP:0003487, HP:0001250, HP:0025047, HP:0005484, HP:0001336, HP:0001337	T+R	27206732
PGAP1	MR079	HP:0000733, HP:0002121, HP:0001319, HP:0001270, HP:0012444, HP:0000400, HP:0001263, HP:0010864, HP:0000252	T+R	24784135
PGAP1	Patient	HP:0012448, HP:0012447, HP:0002079, HP:0012443, HP:0100704, HP:0011968, HP:0100660, HP:0011398, HP:0001263, HP:0000750, HP:0010845, HP:0012429, HP:0000271, HP:0001249	T+R	25823418

Continued on next page

Table **S1** – *Continued from previous page*

Gene	Patient ID	HPOs	Group	PMID
PGAP2	V:6/MR5	HP:0003155, HP:0001270, HP:0002360, HP:0004322, HP:0010864, HP:0001252, HP:0000486, HP:0001324, HP:0002059, HP:0001305, HP:0002536, HP:0003155, HP:0001270, HP:0002360, HP:0004322, HP:0010864, HP:0001252, HP:0000486, HP:0001324, HP:0002059, HP:0001305, HP:0002536, HP:0002121, HP:0001270, HP:0003202, HP:0004322, HP:0010864, HP:0000750, HP:0001324, HP:0002059, HP:0002121, HP:0001270, HP:0003202, HP:0004322, HP:0010864, HP:0000750, HP:0001252, HP:0001324, HP:0002059, HP:0002121, HP:0004322, HP:0002360, HP:0010864, HP:0002059, HP:0002536, HP:0003155, HP:0001270, HP:0003202, HP:0001252, HP:0000750, HP:0000486, HP:0001324, HP:0001305, HP:0002121, HP:0004322, HP:0002360, HP:0010864, HP:0002059, HP:0002536, HP:0003155, HP:0001270, HP:0003202, HP:0001252, HP:0000750, HP:0000486, HP:0001324, HP:0001305	T+R	23561846
PGAP2	IV:1-MR043	HP:0003155, HP:0001270, HP:0002360, HP:0004322, HP:0010864, HP:0001252, HP:0000486, HP:0001324, HP:0002059, HP:0001305, HP:0002536, HP:0003155, HP:0001270, HP:0002360, HP:0004322, HP:0010864, HP:0001252, HP:0000486, HP:0001324, HP:0002059, HP:0001305, HP:0002536	T+R	23561846
PGAP2	Patient 1	HP:0009058, HP:0002100, HP:0010851, HP:0002344, HP:0002521, HP:0030746, HP:0000739, HP:0005469, HP:0003155, HP:0001250, HP:0000767, HP:0001263, HP:0001252, HP:0001265, HP:0003688, HP:0000252, HP:0002093, HP:0003324, HP:0011096, HP:0001946	T+R	26879448
PGAP2	Individual A	HP:0003155, HP:0001270, HP:0001250, HP:0000431, HP:0010804, HP:0001249	T+R	23561847
PGAP2	Individual B	HP:0003155, HP:0001270, HP:0001250, HP:0000431, HP:0010804, HP:0001249, HP:0002251, HP:0002079, HP:0002025, HP:0002650, HP:0000175, HP:0000154, HP:0001631, HP:0000637, HP:0003155, HP:0000316, HP:0001270, HP:0001250, HP:0000455, HP:0001252, HP:0011344, HP:0000750, HP:0000431, HP:0000365, HP:0005484, HP:0010804, HP:0001249	T+R	23561847
PGAP2	Patient 2	HP:0001270, HP:0001250, HP:0001263, HP:0000750, HP:0001510, HP:0000252, HP:0001249, HP:0001270, HP:0001250, HP:0001263, HP:0000750, HP:0001510, HP:0000252, HP:0001249, HP:0003155, HP:0001270, HP:0001250, HP:0001263, HP:0000750, HP:0001510, HP:0000252, HP:0001249, HP:0001270, HP:0001250, HP:0001263, HP:0000750, HP:0001510, HP:0000252, HP:0001249	T+R	27871432
PGAP2	Patient 4-IV-7	HP:0003155, HP:0001263, HP:0000750, HP:0001256, HP:0000805	T+R	29119105
PGAP2	Patient 2-IV-5	HP:0003155, HP:0001250, HP:0001263, HP:0002342, HP:0031491, HP:0000708, HP:0000718	T+R	29119105
PGAP2	Patient 3-IV-6	HP:0003155, HP:0000750, HP:0001575, HP:0001256, HP:0000716	T+R	29119105
PGAP2	Patient 1-IV-3	HP:0002373, HP:0003155, HP:0000750, HP:0001575, HP:0001256, HP:0000716	T+R	29119105

Continued on next page

Table **S1** – *Continued from previous page*

Gene	Patient ID	HPOs	Group	PMID
PGAP3	Patient7-Family7	HP:0002187, HP:0002079, HP:0002069, HP:0002553, HP:0000175, HP:0002265, HP:0000023, HP:0012450, HP:0003194, HP:0000028, HP:0000717, HP:0000637, HP:0000448, HP:0000316, HP:0001270, HP:0003155, HP:0012390, HP:0001250, HP:0003763, HP:0011398, HP:0000431, HP:0000752, HP:0005484, HP:0010804	T+R	28390064
PGAP3	Patient6-Family6	HP:0002079, HP:0012714, HP:0002553, HP:0000175, HP:0010864, HP:0002265, HP:0008501, HP:0002119, HP:0003194, HP:0000717, HP:0000637, HP:0000448, HP:0003155, HP:0000316, HP:0001270, HP:0011398, HP:0000431, HP:0000752, HP:0001344, HP:0005484, HP:0010804, HP:0007930	T+R	28390064
PGAP3	Patient4-Family4	HP:0002079, HP:0002553, HP:0000175, HP:0010864, HP:0002265, HP:0002119, HP:0003194, HP:0000717, HP:0000637, HP:0000448, HP:0003155, HP:0000316, HP:0001270, HP:0000587, HP:0011398, HP:0001321, HP:0000431, HP:0000752, HP:0001344, HP:0005484, HP:0010804	T+R	28390064
PGAP3	Patient5-Family5	HP:0002187, HP:0000060, HP:0002123, HP:0002553, HP:0000175, HP:0002265, HP:0012880, HP:0003194, HP:0000717, HP:0000637, HP:0000448, HP:0003155, HP:0000316, HP:0001270, HP:0000767, HP:0011398, HP:0000431, HP:0001344, HP:0010804	T+R	28390064
PGAP3	Patient8-Family7	HP:0002187, HP:0002079, HP:0002123, HP:0002553, HP:0002265, HP:0002119, HP:0003194, HP:0000717, HP:0030047, HP:0000637, HP:0004325, HP:0000448, HP:0000316, HP:0001270, HP:0003155, HP:0003763, HP:0011398, HP:0000431, HP:0000752, HP:0005484, HP:0001344, HP:0010804, HP:0001357, HP:0001305	T+R	28390064
PGAP3	Patient9-Family8	HP:0002187, HP:0002079, HP:0002069, HP:0002553, HP:0000175, HP:0002265, HP:0003194, HP:0000028, HP:0000717, HP:0000637, HP:0000448, HP:0003155, HP:0000316, HP:0001270, HP:0003763, HP:0011398, HP:0000431, HP:0000752, HP:0001344, HP:0005484, HP:0000540, HP:0010804	T+R	28390064
PGAP3	Patient1-Family1	HP:0002079, HP:0002553, HP:0010864, HP:0002265, HP:0003194, HP:0000639, HP:0000717, HP:0000637, HP:0000448, HP:0003155, HP:0000316, HP:0001270, HP:0000204, HP:0011398, HP:0000202, HP:0001321, HP:0000431, HP:0001344, HP:0005484, HP:0001433, HP:0010804	T+R	28390064
PGAP3	Patient10-Family8	HP:0002079, HP:0001601, HP:0002553, HP:0000175, HP:0010864, HP:0002265, HP:0000023, HP:0003194, HP:0000717, HP:0000637, HP:0000448, HP:0003155, HP:0000316, HP:0001270, HP:0011398, HP:0000431, HP:0000752, HP:0001256, HP:0001344, HP:0000540, HP:0010804	T+R	28390064
PGAP3	Patient2-Family2	HP:0002187, HP:0002079, HP:0002553, HP:0000175, HP:0001643, HP:0002265, HP:0003194, HP:0000717, HP:0000637, HP:0000448, HP:0000316, HP:0001270, HP:0003155, HP:0000822, HP:0003763, HP:0011398, HP:0000202, HP:0000431, HP:0000752, HP:0005484, HP:0001344, HP:0002092, HP:0010804, HP:0010808	T+R	28390064

Continued on next page

Table **S1** – *Continued from previous page*

Gene	Patient ID	HPOs	Group	PMID
PGAP3	Patient3-Family3	HP:0002079, HP:0002069, HP:0002265, HP:0001631, HP:0008501, HP:0003194, HP:0000637, HP:0000448, HP:0001270, HP:0001250, HP:0011398, HP:0003186, HP:0005484, HP:0010804, HP:0010808, HP:0002187, HP:0002553, HP:0006872, HP:0000717, HP:0000316, HP:0003155, HP:0000431, HP:0000752, HP:0001433, HP:0001344	T+R	28390064
PGAP3	V-2-Family A	HP:0002069, HP:0000175, HP:0002265, HP:0004305, HP:0000218, HP:0003155, HP:0000316, HP:0001270, HP:0003196, HP:0000455, HP:0003763, HP:0001263, HP:0001252, HP:0000750, HP:0000431, HP:0005484, HP:0010804, HP:0000582, HP:0010819	T+R	24439110
PGAP3	II-1-Family C	HP:0002079, HP:0002123, HP:0006956, HP:0002265, HP:0003155, HP:0000316, HP:0001270, HP:0003196, HP:0000455, HP:0001252, HP:0011344, HP:0000750, HP:0000431, HP:0000752, HP:0011166, HP:0010804	T+R	24439110
PGAP3	V-3-Family A	HP:0002265, HP:0004305, HP:0000218, HP:0003155, HP:0000316, HP:0001270, HP:0003196, HP:0000455, HP:0003763, HP:0001263, HP:0001252, HP:0011344, HP:0000750, HP:0000431, HP:0005484, HP:0010804, HP:0000582	T+R	24439110
PGAP3	II-1-Family B	HP:0004322, HP:0002123, HP:0002069, HP:0000637, HP:0004325, HP:0003155, HP:0000316, HP:0001270, HP:0001250, HP:0003196, HP:0000455, HP:0001263, HP:0001252, HP:0011344, HP:0000750, HP:0000431, HP:0001344, HP:0010804, HP:0000582	T+R	24439110
PGAP3	V-1-Family A	HP:0002069, HP:0000175, HP:0002265, HP:0004305, HP:0000218, HP:0003155, HP:0000316, HP:0001270, HP:0003196, HP:0000455, HP:0003763, HP:0001263, HP:0001252, HP:0000750, HP:0000431, HP:0005484, HP:0010804, HP:0010819	T+R	24439110
PGAP3	B-II-2	HP:0002123, HP:0002360, HP:0008388, HP:0003155, HP:0000316, HP:0001270, HP:0001251, HP:0003196, HP:0000455, HP:0001263, HP:0001252, HP:0000750, HP:0000431, HP:0000691, HP:0001500, HP:0010804, HP:0002123, HP:0002360, HP:0008388, HP:0003155, HP:0000316, HP:0001270, HP:0001251, HP:0003196, HP:0000455, HP:0001263, HP:0001252, HP:0000750, HP:0000431, HP:0000691, HP:0001500, HP:0010804	T+R	27120253
PGAP3	A-II-1	HP:0002123, HP:0002360, HP:0000175, HP:0000718, HP:0000717, HP:0000637, HP:0000722, HP:0003155, HP:0001270, HP:0410030, HP:0001250, HP:0001263, HP:0000431, HP:0001344, HP:0010804, HP:0000582	T+R	27120253
PGAP3	Younger sister	HP:0002263, HP:0001612, HP:0000175, HP:0008848, HP:0012169, HP:0002098, HP:0000708, HP:0045025, HP:0000319, HP:0004325, HP:0003155, HP:0001263, HP:0000750, HP:0009748, HP:0000278, HP:0000431, HP:0000691, HP:0001344, HP:0005484, HP:0000286	T+R	28794914
PGAP3	Older Brother	HP:0002263, HP:0001999, HP:0002373, HP:0004322, HP:0000175, HP:0002098, HP:0008897, HP:0011968, HP:0000508, HP:0000717, HP:0000319, HP:0001290, HP:0000733, HP:0001270, HP:0001263, HP:0009748, HP:0000431, HP:0000365, HP:0001344, HP:0010804, HP:0000582, HP:0001629, HP:0000490	T+R	28794914

Continued on next page

Table **S1** – *Continued from previous page*

Gene	Patient ID	HPOs	Group	PMID
PGAP3	257982	HP:0012810, HP:0008935, HP:0006108, HP:0002194, HP:0000175, HP:0012345, HP:0025335, HP:0002136, HP:0000219, HP:0000369, HP:0004619, HP:0003186, HP:0000286, HP:0001488, HP:0001357, HP:0001305, HP:0010818, HP:0002121, HP:0002751, HP:0000154, HP:0001763, HP:0000319, HP:0000316, HP:0100543, HP:0003155, HP:0002938, HP:0000403, HP:0000687, HP:0001344	T+R	28327575
PGAP3	257982-affected brother	HP:0012810, HP:0008935, HP:0006108, HP:0002194, HP:0000175, HP:0012345, HP:0025335, HP:0002136, HP:0000219, HP:0000369, HP:0004619, HP:0003186, HP:0000286, HP:0001488, HP:0001357, HP:0001305, HP:0010818, HP:0002121, HP:0000154, HP:0001763, HP:0000319, HP:0000316, HP:0100543, HP:0003155, HP:0002938, HP:0000403, HP:0000687, HP:0001344	T+R	28327575
PGAP3	proband	HP:0001769, HP:0002079, HP:0002360, HP:0006118, HP:0002650, HP:0000175, HP:0002197, HP:0000028, HP:0000736, HP:0001290, HP:0003155, HP:0011443, HP:0000369, HP:0000347, HP:0003763, HP:0001263, HP:0000278, HP:0000431, HP:0010804, HP:0001837	T+R	29531774
PIGT	Patient V-2	HP:0002384, HP:0000072, HP:0006429, HP:0000121, HP:0002750, HP:0000938, HP:0000113, HP:0001290, HP:0005622, HP:0003100, HP:0000248, HP:0000256, HP:0000496, HP:0009824, HP:0000472, HP:0004443, HP:0002650, HP:0010852, HP:0003282, HP:0025330, HP:0000505, HP:0002648, HP:0003022, HP:0001263, HP:0006480, HP:0001520, HP:0000540, HP:0001249, HP:0000271, HP:0100864	T+R	23636107
PIGT	Patient V-1	HP:0006429, HP:0000121, HP:0002750, HP:0000938, HP:0011648, HP:0000639, HP:0004349, HP:0001290, HP:0000767, HP:0000248, HP:0000486, HP:0000256, HP:0009824, HP:0000496, HP:0011330, HP:0002123, HP:0002650, HP:0010852, HP:0100704, HP:0000505, HP:0002648, HP:0001263, HP:0003022, HP:0006480, HP:0001520, HP:0000540, HP:0000271, HP:0001249	T+R	23636107
PIGT	Patient V-5	HP:0000072, HP:0002283, HP:0002069, HP:0000121, HP:0100704, HP:0002750, HP:0004349, HP:0000639, HP:0001272, HP:0000486, HP:0006480, HP:0003186, HP:0009824, HP:0000540, HP:0003429, HP:0001249	T+R	23636107
PIGT	Patient V-4	HP:0007360, HP:0006429, HP:0000072, HP:0002283, HP:0000121, HP:0002750, HP:0000938, HP:0001290, HP:0005622, HP:0003100, HP:0000248, HP:0000256, HP:0003186, HP:0009824, HP:0000496, HP:0000472, HP:0001638, HP:0002123, HP:0012697, HP:0003282, HP:0000505, HP:0002648, HP:0001263, HP:0003022, HP:0006480, HP:0001520, HP:0000540, HP:0000271, HP:0100864, HP:0001249	T+R	23636107

Continued on next page

Gene	Patient ID	HPOs	Group	PMID
PIGT	Patient 2	HP:0009127, HP:0005280, HP:0002069, HP:0010841, HP:0025458, HP:0001631, HP:0002720, HP:0000938, HP:0030718, HP:0002334, HP:0002059, HP:0000639, HP:0000924, HP:0002835, HP:0002714, HP:0002705, HP:0000545, HP:0003100, HP:0011994, HP:0000248, HP:0001250, HP:0001272, HP:0000767, HP:0011996, HP:0000486, HP:0000343, HP:0000365, HP:0000341, HP:0001513, HP:0009131, HP:0001337, HP:0001537, HP:0002187, HP:0002650, HP:0002870, HP:0002020, HP:0002850, HP:0100704, HP:0030856, HP:0030854, HP:0000348, HP:0001382, HP:0001263, HP:0000463, HP:0001336, HP:0010819	T+R	25943031
PIGT	Patient 1	HP:0009127, HP:0005280, HP:0001657, HP:0002069, HP:0010841, HP:0025458, HP:0000938, HP:0002334, HP:0002059, HP:0000639, HP:0002835, HP:0002714, HP:0002705, HP:0003100, HP:0001272, HP:0000248, HP:0000486, HP:0000365, HP:0000343, HP:0000484, HP:0000341, HP:0001929, HP:0005616, HP:0001513, HP:0009131, HP:0003225, HP:0002155, HP:0002650, HP:0002020, HP:0100704, HP:0006254, HP:0000826, HP:0000348, HP:0011167, HP:0000463, HP:0001336	T+R	25943031
PIGT	female proband	HP:0000072, HP:0005280, HP:0011648, HP:0000639, HP:0002059, HP:0008872, HP:0002714, HP:0000218, HP:0000787, HP:0001250, HP:0000347, HP:0000369, HP:0001272, HP:0001252, HP:0001561, HP:0000486, HP:0010804, HP:0000272, HP:0007366, HP:0002187, HP:0000071, HP:0002133, HP:0006913, HP:0002650, HP:0002119, HP:0000939, HP:0003282, HP:0000505, HP:0000463, HP:0000582	T+R	24906948
PIGT	258094	HP:0002373, HP:0006855, HP:0002069, HP:0002073, HP:0002465, HP:0025331, HP:0000505, HP:0001260, HP:0001270, HP:0000787, HP:0001182, HP:0001251, HP:0001272, HP:0001250, HP:0000657, HP:0000455, HP:0001263, HP:0000490	T+R	28327575
PIGT	270250	HP:0002187, HP:0040288, HP:0007141, HP:0001250, HP:0001283, HP:3000033, HP:0200134, HP:0002353, HP:0000252, HP:0003282, HP:0100765, HP:0011471	T+R	28327575
PIGT	270306	HP:0002187, HP:0040288, HP:0007141, HP:0001250, HP:0001283, HP:3000033, HP:0200134, HP:0002353, HP:0000252, HP:0003282, HP:0100765, HP:0011471	T+R	28327575

Table **S2**: GPI-anchored proteins

Protein	Symbol
Acetylcholinesterase (AChE) (EC 3.1.1.7)	ACHE
Intestinal-type alkaline phosphatase (IAP) (Intestinal alkaline phosphatase) (EC 3.1.3.1)	ALPI
Alkaline phosphatase, tissue-nonspecific isozyme (AP-TNAP) (TNSALP) (EC 3.1.3.1) (Alkaline phosphatase liver/bone/kidney isozyme)	ALPL
Alkaline phosphatase, placental type (EC 3.1.3.1) (Alkaline phosphatase Regan isozyme) (Placental alkaline phosphatase 1) (PLAP-1)	ALPP
Alkaline phosphatase, placental-like (EC 3.1.3.1) (ALP-1) (Alkaline phosphatase Nagao isozyme) (Germ cell alkaline phosphatase) (GCAP) (Placental alkaline phosphatase-like) (PLAP-like)	ALPPL2
GPI-linked NAD(P)(+)–arginine ADP-ribosyltransferase 1 (EC 2.4.2.31) (ADP-ribosyltransferase C2 and C3 toxin-like 1) (ARTC1) (Mono(ADP-ribosyl)transferase 1) (CD antigen CD296)	ART1
Ecto-ADP-ribosyltransferase 3 (EC 2.4.2.31) (ADP-ribosyltransferase C2 and C3 toxin-like 3) (ARTC3) (Mono(ADP-ribosyl)transferase 3) (NAD(P)(+)–arginine ADP-ribosyltransferase 3)	ART3
Ecto-ADP-ribosyltransferase 4 (EC 2.4.2.31) (ADP-ribosyltransferase C2 and C3 toxin-like 4) (ARTC4) (Dombrock blood group carrier molecule) (Mono(ADP-ribosyl)transferase 4) (NAD(P)(+)–arginine ADP-ribosyltransferase 4) (CD antigen CD297)	ART4
Brevican core protein (Brain-enriched hyaluronan-binding protein) (BEHAB) (Chondroitin sulfate proteoglycan 7)	BCAN
ADP-ribosyl cyclase/cyclic ADP-ribose hydrolase 2 (EC 3.2.2.6) (ADP-ribosyl cyclase 2) (Bone marrow stromal antigen 1) (BST-1) (Cyclic ADP-ribose hydrolase 2) (cADPr hydrolase 2) (CD antigen CD157)	BST1
Bone marrow stromal antigen 2 (BST-2) (HM1.24 antigen) (Tetherin) (CD antigen CD317)	BST2
Carbonic anhydrase 4 (EC 4.2.1.1) (Carbonate dehydratase IV) (Carbonic anhydrase IV) (CA-IV)	CA4
Calcium channel, voltage-dependent, alpha-2/delta subunit 2	CACNA2D2
CD109 antigen (150 kDa TGF-beta-1-binding protein) (C3 and PZP-like alpha-2-macroglobulin domain-containing protein 7) (Platelet-specific Gov antigen) (p180) (r150) (CD antigen CD109)	CD109
Monocyte differentiation antigen CD14 (Myeloid cell-specific leucine-rich glycoprotein) (CD antigen CD14) [Cleaved into: Monocyte differentiation antigen CD14, urinary form; Monocyte differentiation antigen CD14, membrane-bound form]	CD14
CD160 antigen (Natural killer cell receptor BY55) (CD antigen CD160)	CD160
CD177 antigen (Human neutrophil alloantigen 2a) (HNA-2a) (NB1 glycoprotein) (NB1 GP) (Polycythemia rubra vera protein 1) (PRV-1) (CD antigen CD177)	CD177
Signal transducer CD24 (Small cell lung carcinoma cluster 4 antigen) (CD antigen CD24)	CD24
CD48 antigen (B-lymphocyte activation marker BLAST-1) (BCM1 surface antigen) (Leukocyte antigen MEM-102) (SLAM family member 2) (SLAMF2) (Signaling lymphocytic activation molecule 2) (TCT.1) (CD antigen CD48)	CD48
CAMPATH-1 antigen (CDw52) (Cambridge pathology 1 antigen) (Epididymal secretory protein E5) (Human epididymis-specific protein 5) (He5) (CD antigen CD52)	CD52
Complement decay-accelerating factor (CD antigen CD55)	CD55
Cd58, LFA3, lymphocyte function-associated antigen, type 3	CD58
CD59 glycoprotein (1F5 antigen) (20 kDa homologous restriction factor) (HRF-20) (HRF20) (MAC-inhibitory protein) (MAC-IP) (MEM43 antigen) (Membrane attack complex inhibition factor) (MACIF) (Membrane inhibitor of reactive lysis) (MIRL) (Protectin) (CD antigen CD59)	CD59
Cadherin-13 (Heart cadherin) (H-cadherin) (P105) (Truncated cadherin) (T-cad) (T-cadherin)	CDH13
Carcinoembryonic antigen-related cell adhesion molecule 5 (Carcinoembryonic antigen) (CEA) (Meconium antigen 100) (CD antigen CD66e)	CEACAM5

Continued on next page

Protein	Symbol
Carcinoembryonic antigen-related cell adhesion molecule 6 (Non-specific crossreacting antigen) (Normal cross-reacting antigen) (CD antigen CD66c)	CEACAM6
Carcinoembryonic antigen-related cell adhesion molecule 7 (Carcinoembryonic antigen CGM2)	CEACAM7
Carcinoembryonic antigen-related cell adhesion molecule 8 (CD67 antigen) (Carcinoembryonic antigen CGM6) (Non-specific cross-reacting antigen NCA-95) (CD antigen CD66b)	CEACAM8
Cryptic protein (Cryptic family protein 1)	CFC1
Ciliary neurotrophic factor receptor subunit alpha (CNTF receptor subunit alpha) (CNTFR-alpha)	CNTFR
Contactin-1 (Glycoprotein gp135) (Neural cell surface protein F3)	CNTN1
Contactin-2 (Axonal glycoprotein TAG-1) (Axonin-1) (Transient axonal glycoprotein 1) (TAX-1)	CNTN2
Contactin-3 (Brain-derived immunoglobulin superfamily protein 1) (BIG-1) (Plasmacytoma-associated neuronal glycoprotein)	CNTN3
Contactin-4 (Brain-derived immunoglobulin superfamily protein 2) (BIG-2)	CNTN4
Contactin-5 (Neural recognition molecule NB-2) (hNB-2)	CNTN5
Contactin-6 (Neural recognition molecule NB-3) (hNB-3)	CNTN6
Carboxypeptidase M (CPM) (EC 3.4.17.12)	CPM
Carboxypeptidase O (CPO) (EC 3.4.17.-)	CPO
Dipeptidase (EC 3.4.13.19)	DPEP1
Dipeptidase (EC 3.4.13.19) (Fragment)	DPEP2
Dipeptidase 3 (EC 3.4.13.19)	DPEP3
Ephrin-A1 (EPH-related receptor tyrosine kinase ligand 1) (LERK-1) (Immediate early response protein B61) (Tumor necrosis factor alpha-induced protein 4) (TNF alpha-induced protein 4) [Cleaved into: Ephrin-A1, secreted form]	EFNA1
Ephrin-A2 (EPH-related receptor tyrosine kinase ligand 6) (LERK-6) (HEK7 ligand) (HEK7-L)	EFNA2
Ephrin-A3 (EFL-2) (EHK1 ligand) (EHK1-L) (EPH-related receptor tyrosine kinase ligand 3) (LERK-3)	EFNA3
Ephrin-A4 (EPH-related receptor tyrosine kinase ligand 4) (LERK-4)	EFNA4
Ephrin-A5 (AL-1) (EPH-related receptor tyrosine kinase ligand 7) (LERK-7)	EFNA5
Ectonucleotide pyrophosphatase/phosphodiesterase family member 6 (E-NPP 6) (NPP-6) (EC 3.1.4.-) (EC 3.1.4.38) (Choline-specific glycerophosphodiester phosphodiesterase) (Glycerophosphocholine cholinephosphodiesterase) (GPC-Cpde)	ENPP6
Low affinity immunoglobulin gamma Fc region receptor III-B (Fc-gamma RIII-beta) (Fc-gamma RIII) (Fc-gamma RIIIb) (FcRIII) (FcRIIIb) (FcR-10) (IgG Fc receptor III-1) (CD antigen CD16b)	FCGR3B
Folate receptor alpha (FR-alpha) (Adult folate-binding protein) (FBP) (Folate receptor 1) (Folate receptor, adult) (KB cells FBP) (Ovarian tumor-associated antigen MOv18)	FOLR1
Folate receptor beta (FR-beta) (Folate receptor 2) (Folate receptor, fetal/placental) (Placental folate-binding protein) (FBP)	FOLR2
Folate receptor gamma (FR-gamma) (Folate receptor 3)	FOLR3
Growth arrest-specific protein 1 (GAS-1)	GAS1
GDNF family receptor alpha-1 (GDNF receptor alpha-1) (GDNFR-alpha-1) (GFR-alpha-1) (RET ligand 1) (TGF-beta-related neurotrophic factor receptor 1)	GFRA1
GDNF family receptor alpha-2 (GDNF receptor alpha-2) (GDNFR-alpha-2) (GFR-alpha-2) (GDNF receptor beta) (GDNFR-beta) (Neurturin receptor alpha) (NRTNR-alpha) (NTNR-alpha) (RET ligand 2) (TGF-beta-related neurotrophic factor receptor 2)	GFRA2
GDNF family receptor alpha-3 (GDNF receptor alpha-3) (GDNFR-alpha-3) (GFR-alpha-3)	GFRA3
GDNF family receptor alpha-4 (GDNF receptor alpha-4) (GDNFR-alpha-4) (GFR-alpha-4) (Persephin receptor)	GFRA4
GLIPR1-like protein 1	GLIPR1L1
Glycosyl-phosphatidylinositol-anchored molecule-like protein	GML

Continued on next page

Table **S2** – *Continued from previous page*

Protein	Symbol
Pancreatic secretory granule membrane major glycoprotein GP2 (Pancreatic zymogen granule membrane protein GP-2) (ZAP75)	GP2
Glypican-1 [Cleaved into: Secreted glypican-1]	GPC1
Glypican-2 [Cleaved into: Secreted glypican-2]	GPC2
Glypican-3 (Fragment)	GPC3
K-glypican	GPC4
Glypican-5 [Cleaved into: Secreted glypican-5]	GPC5
Glypican-6 [Cleaved into: Secreted glypican-6]	GPC6
Glycosylphosphatidylinositol-anchored high density lipoprotein-binding protein 1 (GPI-HBP1) (GPI-anchored HDL-binding protein 1) (High density lipoprotein-binding protein 1)	GPIHBP1
Hemojuvelin (Hemochromatosis type 2 protein) (RGM domain family member C)	HFE2
Hyaluronidase-2 (Hyal-2) (EC 3.2.1.35) (Hyaluronoglucosaminidase-2) (Lung carcinoma protein 2) (LuCa-2)	HYAL2
Immunoglobulin superfamily member 21 (IgSF21)	IGSF21
Intelectin-1 (ITLN-1) (Endothelial lectin HL-1) (Galactofuranose-binding lectin) (Intestinal lactoferrin receptor) (Omentin)	ITLN1
Sperm-egg fusion protein Juno (Folate receptor 4) (Folate receptor delta) (FR-delta) (IZUMO1 receptor protein JUNO)	IZUMO1R
Lipoprotein lipase (LPL) (EC 3.1.1.34)	LPL
Limbic system-associated membrane protein (LSAMP) (IgLON family member 3)	LSAMP
Lymphocyte antigen 6D (Ly-6D) (E48 antigen)	LY6D
Lymphocyte antigen 6E (Ly-6E) (Retinoic acid-induced gene E protein) (RIG-E) (Stem cell antigen 2) (SCA-2) (Thymic shared antigen 1) (TSA-1)	LY6E
Lymphocyte antigen 6 complex locus protein G6c	LY6G6C
Lymphocyte antigen 6 complex locus protein G6d (Protein Ly6-D) (Megakaryocyte-enhanced gene transcript 1 protein)	LY6G6D
Lymphocyte antigen 6H (Ly-6H)	LY6H
Lymphocyte antigen 6K (Ly-6K)	LY6K
Lymphocyte antigen 6L (Lymphocyte antigen 6 complex locus protein L)	LY6L
Ly-6/neurotoxin-like protein 1 (Endogenous prototoxin LYNX1) (Testicular tissue protein Li 112)	LYNX1
Ly6/PLAUR domain-containing protein 1 (Putative HeLa tumor suppressor) (PHTS)	LYPD1
Ly6/PLAUR domain-containing protein 2	LYPD2
Ly6/PLAUR domain-containing protein 3 (GPI-anchored metastasis-associated protein C4.4A homolog) (Matrigel-induced gene C4 protein) (MIG-C4)	LYPD3
Ly6/PLAUR domain-containing protein 4	LYPD4
Ly6/PLAUR domain-containing protein 5	LYPD5
Ly6/PLAUR domain-containing protein 6	LYPD6
Ly6/PLAUR domain-containing protein 6B	LYPD6B
Ly6/PLAUR domain-containing protein 8	LYPD8
MAM domain-containing glycosylphosphatidylinositol anchor protein 1 (Fragment)	MDGA1
MAM domain-containing glycosylphosphatidylinositol anchor protein 2	MDGA2
Melanotransferrin (Melanoma-associated antigen p97) (CD antigen CD228)	MELTF
Matrix metalloproteinase-17 (MMP-17) (EC 3.4.24.-) (Membrane-type matrix metalloproteinase 4) (MT-MMP 4) (MTMMP4) (Membrane-type-4 matrix metalloproteinase) (MT4-MMP) (MT4MMP)	MMP17
Matrix metalloproteinase-25 (MMP-25) (EC 3.4.24.-) (Leukolysin) (Membrane-type matrix metalloproteinase 6) (MT-MMP 6) (MTMMP6) (Membrane-type-6 matrix metalloproteinase) (MT6-MMP) (MT6MMP)	MMP25
Mesothelin (CAK1 antigen) (Pre-pro-megakaryocyte-potentiating factor) [Cleaved into: Megakaryocyte-potentiating factor (MPF); Mesothelin, cleaved form]	MSLN
Neural cell adhesion molecule 1 (N-CAM-1) (NCAM-1) (CD antigen CD56)	NCAM1
Neuronal growth regulator 1 (IgLON family member 4)	NEGR1
Neuritin	NRN1
Neuritin-like protein	NRN1L
5'-nucleotidase (5'-NT) (EC 3.1.3.5) (Ecto-5'-nucleotidase) (CD antigen CD73)	NT5E
Neurotrimin (hNT) (IgLON family member 2)	NTM

Continued on next page

Protein	Symbol
Netrin-G1 (Laminet-1)	NTNG1
Netrin-G2 (Laminet-2)	NTNG2
Oligodendrocyte-myelin glycoprotein	OMG
Opioid-binding protein/cell adhesion molecule (OBCAM) (OPCML) (Opioid-binding cell adhesion molecule) (IgLON family member 1)	OPCML
Otoancorin	OTOA
Urokinase plasminogen activator surface receptor (U-PAR) (uPAR) (Monocyte activation antigen Mo3) (CD antigen CD87)	PLAUR
Placenta-expressed transcript 1 protein	PLET1
Prion-like protein doppel (PrPLP) (Prion protein 2)	PRND
Major prion protein (PrP) (ASCR) (PrP27-30) (PrP33-35C) (CD antigen CD230)	PRNP
Testisin (EC 3.4.21.-) (Eosinophil serine protease 1) (ESP-1) (Serine protease 21)	PRSS21
Serine protease 41 (EC 3.4.21.-) (Testis serine protease 1) (TESSP-1)	PRSS41
Prostate stem cell antigen	PSCA
UL-16 binding protein 5 (Retinoic acid early transcript 1G protein)	RAET1G
UL16-binding protein 6 (Retinoic acid early transcript 1L protein)	RAET1L
Reversion-inducing cysteine-rich protein with Kazal motifs (hRECK) (Suppressor of tumorigenicity 15 protein)	RECK
Repulsive guidance molecule A (RGM domain family member A)	RGMA
RGM domain family member B (DRG11-responsive axonal guidance and outgrowth of neurite) (DRAGON)	RGMB
Reticulon-4 receptor (Nogo receptor) (NgR) (Nogo-66 receptor)	RTN4R
Reticulon-4 receptor-like 1 (Nogo receptor-like 2) (Nogo-66 receptor homolog 2) (Nogo-66 receptor-related protein 3) (NgR3)	RTN4RL1
Reticulon-4 receptor-like 2 (Nogo receptor-like 3) (Nogo-66 receptor homolog 1) (Nogo-66 receptor-related protein 2) (NgR2)	RTN4RL2
Semaphorin-7A (CDw108) (JMH blood group antigen) (John-Milton-Hargen human blood group Ag) (Semaphorin-K1) (Sema K1) (Semaphorin-L) (Sema L) (CD antigen CD108)	SEMA7A
Acid sphingomyelinase-like phosphodiesterase 3b (ASM-like phosphodiesterase 3b) (EC 3.1.4.-)	SMPDL3B
Sperm acrosome membrane-associated protein 4 (Sperm acrosomal membrane-associated protein 14)	SPACA4
Hyaluronidase PH-20 (Hyal-PH20) (EC 3.2.1.35) (Hyaluronoglucosaminidase PH-20) (Sperm adhesion molecule 1) (Sperm surface protein PH-20)	SPAM1
Shadow of prion protein (Protein shadoo)	SPRN
Teratocarcinoma-derived growth factor 1 (Cripto-1 growth factor) (CRGF) (Epidermal growth factor-like cripto protein CR1)	TDGF1
Alpha-tectorin	TECTA
Beta-tectorin	TECTB
Testis-expressed protein 101 (Cell surface receptor NYD-SP8) (Scleroderma-associated autoantigen) (Spermatogenesis-related gene protein)	TEX101
Tissue factor pathway inhibitor (TFPI) (Extrinsic pathway inhibitor) (EPI) (Lipoprotein-associated coagulation inhibitor) (LACI)	TFPI
Thy-1 membrane glycoprotein (CDw90) (Thy-1 antigen) (CD antigen CD90)	THY1
Tumor necrosis factor receptor superfamily member 10C (Antagonist decoy receptor for TRAIL/Apo-2L) (Decoy TRAIL receptor without death domain) (Decoy receptor 1) (DcR1) (Lymphocyte inhibitor of TRAIL) (TNF-related apoptosis-inducing ligand receptor 3) (TRAIL receptor 3) (TRAIL-R3) (TRAIL receptor without an intracellular domain) (CD antigen CD263)	TNFRSF10C
Trehalase (EC 3.2.1.28) (Alpha,alpha-trehalase) (Alpha,alpha-trehalose glucohydrolase)	TREH
UL16-binding protein 1 (ALCAN-beta) (NKG2D ligand 1) (N2DL-1) (NKG2DL1) (Retinoic acid early transcript 1I)	ULBP1
UL16-binding protein 2 (ALCAN-alpha) (NKG2D ligand 2) (N2DL-2) (NKG2DL2) (Retinoic acid early transcript 1H)	ULBP2
UL16-binding protein 3 (ALCAN-gamma) (NKG2D ligand 3) (N2DL-3) (NKG2DL3) (Retinoic acid early transcript 1N)	ULBP3

Continued on next page

Table **S2** – *Continued from previous page*

Protein	Symbol
Uromodulin (Tamm-Horsfall urinary glycoprotein) (THP) [Cleaved into: Uromodulin, secreted form]	UMOD
Pantetheinase (EC 3.5.1.92) (Pantetheine hydrolase) (Tiff66) (Vascular non-inflammatory molecule 1) (Vanin-1)	VNN1
Vascular non-inflammatory molecule 2 (Vanin-2) (EC 3.5.1.92) (Glycosylphosphatidyl inositol-anchored protein GPI-80) (Protein FOAP-4)	VNN2
Vascular non-inflammatory molecule 3 (Vanin-3) (EC 3.5.1.92)	VNN3
Xaa-Pro aminopeptidase 2 (EC 3.4.11.9) (Aminoacylproline aminopeptidase) (Membrane-bound aminopeptidase P) (Membrane-bound APP) (Membrane-bound AmP) (mAmP) (X-Pro aminopeptidase 2)	XPNPEP2

Table **S3**: Phenotypic comparison of Synthesis+Transamidase (S/T) vs. Remodeling groups

HPO ID	HPO label	S/T	Remodelling	χ^2	p-val (adj)
HP:0000079	*Abnormality of the urinary system*	35/113 (31.0%)	1/39 (2.6%)	12.946	0.04648
HP:0010935	*Abnormality of the upper urinary tract*	34/113 (30.1%)	0/39 (0.0%)	15.116	0.01466
HP:0000708	*Behavioral abnormality*	15/113 (13.3%)	24/39 (61.5%)	35.407	0
HP:0012758	*Neurodevelopmental delay*	72/113 (63.7%)	39/39 (100.0%)	19.377	0.00156
HP:0000750	*Delayed speech and language development*	34/113 (30.1%)	30/39 (76.9%)	26.089	0.00005
HP:0001270	*Motor delay*	22/113 (19.5%)	29/39 (74.4%)	39.181	0
HP:0040195	*Decreased head circumference*	21/113 (18.6%)	19/39 (48.7%)	13.577	0.03319
HP:0000252	*Microcephaly*	20/113 (17.7%)	19/39 (48.7%)	14.625	0.01902
HP:0005484	*Postnatal microcephaly*	13/113 (11.5%)	16/39 (41.0%)	16.366	0.00757
HP:0000598	*Abnormality of the ear*	27/113 (23.9%)	22/39 (56.4%)	14.033	0.02604
HP:0031703	*Abnormal ear morphology*	21/113 (18.6%)	21/39 (53.8%)	18.029	0.00316
HP:0000356	*Abnormality of the outer ear*	18/113 (15.9%)	21/39 (53.8%)	21.853	0.00043
HP:0000377	*Abnormality of the pinna*	17/113 (15.0%)	18/39 (46.2%)	15.831	0.01004
HP:0100886	*Abnormality of globe location*	26/113 (23.0%)	22/39 (56.4%)	14.971	0.01583
HP:0000316	*Hypertelorism*	23/113 (20.4%)	20/39 (51.3%)	13.671	0.03158
HP:0030669	*Abnormal morphology of the ocular adnexa*	20/113 (17.7%)	21/39 (53.8%)	19.232	0.00168
HP:0000492	*Abnormal eyelid morphology*	19/113 (16.8%)	20/39 (51.3%)	18.058	0.00311
HP:0008050	*Abnormality of the palpebral fissures*	13/113 (11.5%)	18/39 (46.2%)	21.44	0.00053
HP:0200007	*Abnormal size of the palpebral fissures*	7/113 (6.2%)	14/39 (35.9%)	21.483	0.00052
HP:0000422	*Abnormality of the nasal bridge*	23/113 (20.4%)	22/39 (56.4%)	18.086	0.00306
HP:0000431	*Wide nasal bridge*	13/113 (11.5%)	22/39 (56.4%)	32.987	0
HP:0000159	*Abnormality of the lip*	32/113 (28.3%)	24/39 (61.5%)	13.751	0.03027
HP:0000177	*Abnormality of upper lip*	28/113 (24.8%)	24/39 (61.5%)	17.407	0.00437
HP:0011339	*Abnormality of upper lip vermillion*	23/113 (20.4%)	24/39 (61.5%)	23.023	0.00023
HP:0010804	*Tented upper lip vermilion*	20/113 (17.7%)	21/39 (53.8%)	19.232	0.00168
HP:0100737	*Abnormality of the hard palate*	6/113 (5.3%)	17/39 (43.6%)	33.084	0
HP:0000202	*Oral cleft*	6/113 (5.3%)	18/39 (46.2%)	36.377	0
HP:0000175	*Cleft palate*	6/113 (5.3%)	17/39 (43.6%)	33.084	0
HP:0001939	*Abnormality of metabolism/homeostasis*	54/113 (47.8%)	32/39 (82.1%)	13.855	0.02863
HP:0012379	*Abnormal enzyme/coenzyme activity*	37/113 (32.7%)	31/39 (79.5%)	25.624	0.00006
HP:0004379	*Abnormality of alkaline phosphatase activity*	37/113 (32.7%)	31/39 (79.5%)	25.624	0.00006
HP:0003155	*Elevated alkaline phosphatase*	32/113 (28.3%)	31/39 (79.5%)	31.28	0

Table **S4**: Publications curated for this work

Author	Title	Year	PMID
Abdel-Hamid	PGAP3-related hyperphosphatasia with mental retardation syndrome: Report of 10 new patients and a homozygous founder mutation.	2017	28390064
Almeida	Hypomorphic promoter mutation in PIGM causes glycosylphophatidylintosol deficiency	2006	16767100
Barone	DPM2-CDG: a muscular dystrophy-dystroglycanopathy syndrome with severe epilepsy.	2012	23109149
Belet	Early frameshift mutation in PIGA identified a large XLID family without neonatal lethality.	2014	24357517
Bosch	Cerebral visual impairment and intellectual disability caused by PGAP1 variants.	2015	25804403
Brady	Exome sequencing identifies a recessive PIGN splice site mutation as a cause of syndromic congentital diaphragmatic hernia	2014	24852103
Ceroni JR1	Large deletion in PIGL: a common mutational mechanism in CHIME syndrome?	2018	29473937
Chiyonobu	Glycosylphophatidylinositol (GPI) anchor deficiency caused by mutations in PIGW is associated with WEST syndrome and hyperphosphatasia with mental retardation syndrome	2014	24367057
Edvarson	Mutations in the phosphatidylinositol glycan C (PIGC) gene are associated with epilepsy and intellectual disability.	2017	27694521
Fujiwara	Mutations in PIGL in a patient with Mabry syndrome	2015	
Granzow	Loss of function of PGAP1 as a cause of severe encephalopathy identified by Whole Exome Sequencing: Lessons of the bioinformatics pipeline.	2015	26050939
Hansen	Hypomorphic mutations in PGAP2, encoding a GPI-anchor-remodeling protein, cause autosomal-recessive intellectual disability.	2013	23561846
Hogrebe	A novel mutation in PIGW causes glycosylphosphatidylinositol deficiency without hyperphosphatasia	2016	27626616
Horn	Delineation of PIGV mutation spectrum and associated phenotypes in hyperphosphatasia with mental retardation syndrome	2014	24129430
Howard	Mutations in PGAP3 impair GPI-anchor maturation, causing a subtype of hyperphosphatasia with mental retardation.	2014	24439110
Ilkovski	Mutations in PIGY: expanding the phenotype of inherited glycosylphophatidylinosol deficiencies	2015	26293662
Jezela-Stanek	Congenital disorder of glycosylphosphatidylinositol (GPI)-anchor biosynthesis–The phenotype of two patients with novel mutations in the PIGN and PGAP2 genes	2016	26879448
Johnston	The phenotype of a germline mutation in PIGA: the gene somatically mutated paroxysmal nocturnal hemoglobinuria	2012	22305531
Johnstone	Compound heterozygous mutations in the gene PIGP are associated with early infantile epileptic encephalopathy.	2017	28334793
Kato	PIGA mutations cause early onset epileptic encephalopathies and distinctive features	2014	24706016

Continued on next page

Table **S4** – *Continued from previous page*

Author	Title	Year	PMID
Kettwig	Compound heterozygous variants in PGAP1 causing severe psychomotor retardation, brain atrophy, recurrent apneas and delayed myelination: a case report and literature review	2016	27206732
Kim	Dolichol phosphate mannose synthase (DPM1) mutations define congenital disorder of glycosylation Ie (CDG-Ie)	2000	10642597
Knaus	Rare Noncoding Mutations Extend the Mutational Spectrum in the PGAP3 Subtype of Hyperphosphatasia with Mental Retardation Syndrome.	2016	27120253
Krawitz	PGAP2 mutations, affecting the GPI-anchor-synthesis pathway, cause hyperphosphatasia with mental retardation syndrome	2013	23561847
Krawitz	Mutations in PIGO, a member of the GPI-anchor-synthesis pathway, cause hyperphosphatasia with mental retardation	2012	
Kuki	B6-responsive epilepsy due to inherited GPI deficiency	2013	24049131
Kvamung	A novel intellectual disabilitysyndrome caused by GPI anchor deficiency due to homozygous mutations in PIGT	2013	23636107
Lam	Expanding the clinical and molecular characteristics of PIGT-CDG, a disorder of glycosylphosphatidylinositol anchors.	2015	25943031
Lefebeder	Deficiency of Dol-P-Man synthase subunit DPM3 bridges the congenital disorders of glycosylation with the dystroglycanopathies.	2009	19576565
Lin	We report the first family with PIGA-associated epileptic encephalopathy in Taiwan and hope to elucidate its special phenotype and inheritance pattern.	2018	29656098
Makrythanasis	Pathogenic Variants in PIGG Cause Intellectual Disability with Seizures and Hypotonia	2016	26996948
Martin	Clinical whole=genome sequencing in severe early-onset epilepsy reveals new genes and improves molecular diagnosis	2014	
Maydan	Multiple congenital anomalies-hypotonia-seizures syndrome is caused by a mutation in PIGN	2011	21493957
McInerney-Leo	Fryns Syndrome Associated with Recessive Mutations in PIGN in two Separate Families.	2016	27038415
Murakami	Null mutation in PGAP1 impairing Gpi-anchor maturation in patients with intellectual disability and encephalopathy.	2014	24784135
Nakamura	PIGO mutations in intractable epilepsy and sever developmental delay with mild elevation of alkaline phosphatse levels	2014	24417746
Nakashima	Novel coumpund heterozygous PIGT mutations caused multiple congenital anomalies-hypotonia seizures syndrome 3	2014	24906948
Nampoothiri	Hyperphosphatasia with Mental Retardation Syndrome Due to a Novel Mutation in PGAP3	2017	28794914
Naseer	A novel mutation in PGAP2 gene causes developmental delay, intellectual disability, epilepsy and microcephaly in consanguineous Saudi family.	2016	27871432
Ng	Mutations in the glycosylphosphatidylinositol gene PIGL cause CHIME syndrome.	2012	
Nguyen	Mutations in GPAA1, Encoding a GPI Transamidase Complex Protein, Cause Developmental Delay, Epilepsy, Cerebellar Atrophy, and Osteopenia.	2017	29100095

Continued on next page

Table **S4** – *Continued from previous page*

Author	Title	Year	PMID
Nguyen	A PIGH mutation leading to GPI deficiency is associated with developmental delay and autism.	2018	29603516
Ohba	PIGN mutations cause congenital anomalies, developmental delay, hypotonia, epilepsy, and progressive cerebellar atrophy	2014	24253414
Pagnamenta	A homozygous variant disrupting the PIGH start-codon is associated with developmental delay, epilepsy, and microcephaly.	2018	PMID: 29573052
Pagnamenta	Analysis of exome data for 4293 trios suggests GPI-anchor biogenesis defects are a rare cause of developmental disorders.	2017	28327575
Perez	A Rare Variant in PGAP2 Causes Autosomal Recessive Hyperphosphatasia with Mental Retardation Syndrome, with a Mild Phenotype in Heterozygous Carriers.	2017	29119105
Sakaguchi	A novel PGAP3 mutation in a Croatian boy with brachytelephalangy and a thin corpus callosum.	2017	29531774
Schenk	MPDU1 mutations underlie a novel human congenital disorder of glycosylation, designated type If.	2001	11733564
Swoboda	A novel germline PIGA mutation in Ferro-Cerebro-Cutaneous syndrome: a neurodegenerative X-linked epileptic encephalopathy with systemic iron-overload	2014	24259288
Tarailo-Graovac	The genotypic and phenotypic spectrum of PIGA deficiency	2015	
Thiffault	Hypotonia and intellectual disability without dysmorphic features in a patient with PIGN-related disease	2017	29096607
Thompson	Phenotypic variability in hyperphosphatasia with seizures and neurologic deficit (Mabry syndrome).	2012	22315194
Van den Bergh	A homozygous DPM3 mutation in a patient with alpha-dystroglycan-related limb girdle muscular dystrophy	2017	28803818
Van der Crabben	Expanding the spectrum of phenotypes associated with germline PIGA mutations: a child with developmental delay, accelerated linear growth, facial dysmorphisms, elevated alkaline phosphatase, and progressive CNS abnormalities	2014	24259184
Williams	Additional evidence that PGAP1 loss of function causes autosomal recessive global developmental delay and encephalopathy.	2015	25823418
Xie LL	A novel germline PIGA mutation causes early-onset epileptic encephalopathies in Chinese monozygotic twins.	2018	29502866
Yang	Congenital disorder of glycosylation due to DPM1 mutations presenting with dystroglycanopathy-type congenital muscular dystrophy.	2013	23856421
Zehavi	A homozygous PIGO mutation associated with severe infantile epileptic encephalopathy and corpus callosum hypoplasia, but normal alkaline phosphatase levels.	2017	28900819
